# Carbon nanomaterials: Exploring new frontiers in wound healing therapy

**DOI:** 10.1002/btm2.70071

**Published:** 2025-10-14

**Authors:** Pegah Madaninasab, Mahsa Mohammadzadeh, Sheyda Labbaf

**Affiliations:** ^1^ Department of Materials Engineering Isfahan University of Technology Isfahan Iran

**Keywords:** biomedical applications, carbon nanomaterials, e‐skin, therapeutic potential, wound healing

## Abstract

This comprehensive review explores the therapeutic potential of carbon nanomaterials, including carbon nanotubes, graphene, carbon dots, and other related materials, in wound healing applications. These materials offer a cutting‐edge approach by modulating critical cellular processes, addressing current challenges in wound care, and advancing tissue regeneration techniques. The article thoroughly examines recent developments in carbon nanomaterials, highlighting their integration into wound care strategies and the ongoing efforts to overcome limitations such as biocompatibility, toxicity, and long‐term safety. Unlike previous reviews, this work not only acknowledges recent advancements but also provides a critical analysis of the still existing barriers and novel strategies for effectively translating these materials from research to clinical applications. By emphasizing both the potential and the challenges, the review aims to present a unique perspective on the future of carbon nanomaterials in wound healing, paving the way for more efficient and personalized treatment options.

## Graphical abstract

The skin is the body's main defense barrier, making it susceptible to all kinds of wounds. Various dressings have been used to improve the skin, and this review article discusses recent progress in carbon‐based nanomaterials, addressing their challenges, potential solutions, and future prospects of use in promoting skin regeneration.

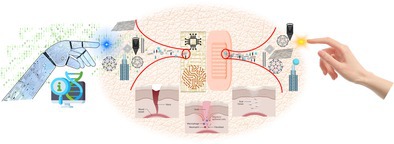

**Abbreviations**
ACF‐CNFs: activated carbon micro‐nanofibers
AgNP: silver nanoparticle
AI: artificial intelligence
AI‐HTPCSS: artificial intelligence‐assisted high throughput printing‐condition‐screening system
AiIoMT: Combination of Internet of Medical Things and Artificial intelligence
AlgMA: sodium methacrylate‐alginate
ANOVA: analysis of variance
CA: cellulose acetate
CAP: cellulose acetate phthalate
CD3: cluster of differentiation 3
CD31: cluster of differentiation 31
CD4: cluster of differentiation 4
CDs: carbon‐based dots
CFs: carbon fibers
Cmap: connection map
CMGG: carboxymethyl guar gum
CNDs: carbon nanodot
CNFs: carbon nanofibers
CNOs: carbon nano‐onions
CNTs: carbon nanotubes
CPGH: fluorene‐polydopamine‐gelatin methacrylate hydrogel or C60‐PDA/GelMA hydrogel
CQDs: carbon quantum dots
CS: chitosan
Cu‐NPs: copper nanoparticles
Cur: curcumin
CV: coefficient of variations
CVD: chemical vapor deposition
DM: diabetes mellitus control (non‐treatment)
DMF: dimethylformamide
DMSO: dimethyl sulfoxide
DPPH: 2,2‐diphenyl‐1‐picrylhydrazyl free radical
DWCNTs: double‐walled carbon nanotubes
EA.hy 926 endothelial cells: immortalized human vascular endothelial cells
*E. coli*
: *Escherichia coli*
ECM: extracellular matrix
ELISA: enzyme‐linked immunoassay
eNOS: endothelial nitric oxide synthase
E‐skin: electronic skin
FDA: Food and Drug Administration
GelMA: gelatin methacrylate
GG: gellan gam
GH: gelatin methacrylate hydrogel or GelMA hydrogel
GNS: graphene nanosheets
GO: graphene oxide
GONWs: graphene oxide nanowalls
GQDs: graphene quantum dots
GSDMDN: Gasdermin D
HaCaT: human epidermal keratinocyte cells
HDAC4: histone deacetylase 4
hDFs: human dermal fibroblast cells
H&E: hematoxylin and eosin
HPHT: high pressure and high temperature
IL‐6: interleukin 6
IL‐1α: interleukin‐1 alpha
IL‐1β: interleukin‐1 beta
ISO: International Organization for Standardization
MC3T3‐E1 cells: osteoblastic cell line
MMP 9: matrix metalloproteinase‐9
MN: microneedle
MRSA: methicillin‐resistant *Staphylococcus aureus*
MW: microwave
MWCNTs: multi‐walled carbon nanotubes
NDs: nanodiamond particles
nFC: nanofibril cellulose
NHDF cells: primary normal human dermal fibroblasts cells
NIH3T3: embryonic mouse fibroblast cell line
NIR: near‐infrared radiation
NPs: nanoparticles
NV−: negatively charged nitrogen‐vacancy
OD: optical density
ODMR: optical magneto‐resonance
OECD: Organisation for Economic Co‐operation and Development
PA6: polyamide 6
PBS: phosphate‐buffered saline
PCL: polycaprolactone
PCL‐CA: polycaprolactone‐cellulose acetate
PDA: polydopamine
PDMS: polydimethylsiloxane
PNIPAm: poly *N*‐isopropioacrylamide
PTFE: polytetrafluoroethylene
PVA: polyvinyl alcohol
QCS: quaternary chitosan
Raw 264.7: macrophage cell line
rGO: reduced graphene oxide
RGNWs: reduced graphene oxide nanowalls
ROS: reactive oxygen species
*S. aureus*
: *Staphylococcus aureus*
SD: standard deviation
SEM: scanning electron microscope
SWCNTs: single‐walled carbon nanotubes
TA: tannin
TEM: transmission electron microscope
TES‐PAMAM‐G3: triethoxysilane poly(amidoamine) dendrimer generation 3
TGA: thermogravimetric analyzer
TGF‐β1: transforming growth factor beta 1
THF: tetrahydrofuran
TNF‐α: tumor necrosis factor‐alpha
TSA: trichostatin A
UVB: ultraviolet B radiation
VEGFA: vascular endothelial growth factor A
VEGF‐a: vascular endothelial growth factor a
WB: Western Blotting
YE: yeast
ZnONP: zinc oxide nanoparticles

**Translational Impact Statement**

Recent advances in carbon‐based nanomaterial therapeutics have demonstrated significant potential in enhancing wound care by facilitating beneficial cellular and biological responses essential for tissue repair and regeneration. The distinctive physicochemical properties of these materials position them as promising candidates for next‐generation therapeutic platforms. The future aims to combine smart technologies and personalized care with nanotechnology, especially carbon nanomaterials, leveraging their unique properties. However, challenges in accurately monitoring their function in the body and concerns about long‐term biosafety have slowed their clinical applications. These challenges will require coordinated efforts among researchers, industry, and regulatory bodies to ensure the safe and effective translation of carbon nanomaterials into clinical applications.

## 1 INTRODUCTION

Wound healing is a complex biological process involving a series of intricately orchestrated events to restore tissue integrity and functionality.^1^, ^2^, ^3^ The ability to control and expedite this complicated cascade of events has long been a crucial point in medical research, as efficient wound healing is crucial for preventing infections, minimizing scar formation, and restoring tissue homeostasis.^4^, ^5^, ^6^ The intricate sequence involves hemostasis, inflammation, proliferation, and tissue remodeling, all orchestrated by a delicate interplay of cells, growth factors, and extracellular matrix (ECM) components.^2^, ^7^, ^8^ Despite significant progress in understanding the molecular and cellular mechanisms underlying wound healing, there remains a persistent need for innovative therapeutic strategies that can enhance and accelerate the regenerative process.

In recent years, nanotechnology has emerged as a promising frontier in wound healing, offering innovative solutions to address the limitations of conventional approaches.^9^, ^10^, ^11^, ^12^, ^13^ The unique properties of nanomaterials, such as their high surface area‐to‐volume ratio, tunable physicochemical characteristics, and the ability to interact at the molecular and cellular levels, make them ideal candidates for targeted and controlled therapeutic interventions.^14^, ^15^ Among the many types of nanomaterials, carbon‐based nanomaterials stand out for their exceptional mechanical strength, high surface area, and favorable biocompatibility.^16^, ^17^, ^18^ The distinctive properties of carbon nanotubes, graphene, carbon dots, and other related materials make them compelling candidates for various biomedical applications, including drug delivery,^19^, ^20^ imaging,^21^, ^22^, ^23^ tissue engineering,^24^, ^25^ and, notably, wound healing.^26^, ^27^

This comprehensive review aims to present a novel and differentiated perspective on the therapeutic potential of carbon nanomaterials in wound healing, not only by elucidating their underlying mechanisms and recent advancements but also by critically analyzing the current challenges that hinder their clinical application. Unlike previous reviews, which predominantly focus on general applications of carbon nanomaterials, this review emphasizes their specific and groundbreaking role in wound treatment, with an in‐depth analysis of their impact on vital cellular processes such as inflammation, cell migration, proliferation, and ECM remodeling. A key innovation of this review lies in its critical evaluation of unresolved challenges—such as biocompatibility, toxicity, and long‐term safety—offering new insights and strategies to overcome these barriers. Additionally, this review addresses the current unmet clinical need for novel methods to fully harness the potential of carbon nanomaterials in clinical wound care. By integrating both recent advancements and a forward‐looking perspective, this review provides a comprehensive roadmap for future research, positioning carbon nanomaterials as revolutionary agents in personalized wound healing therapies.

## 2 WOUNDS

Wounds, ranging from acute injuries to chronic ulcers and those resulting from surgical procedures, represent a significant medical challenge necessitating thorough exploration of innovative therapeutic avenues.^4^, ^28^, ^29^, ^30^ This section provides a comprehensive understanding of wound types, encompassing acute injuries, chronic wounds, and those arising from surgical interventions. We investigate the categorization of wounds, examining their distinctive characteristics and clinical implications. A critical evaluation of traditional treatment methods is presented, offering an overview of conventional wound care approaches that have long been widely used in clinical practice. Despite the progress made in the field, challenges persist in current wound care practices, with limitations existing in the efficacy of conventional treatments. Recognizing the need for transformative approaches, this section explores modern treatment methods utilized in nanotechnology. The integration of nanotechnology in current wound treatments represents a paradigm shift, holding the promise of addressing the limitations of conventional strategies.^11^, ^31^, ^32^, ^33^ As we navigate through this section, we aim to highlight the progress in wound management, emphasizing the unique role of nanotechnology in shaping the future of wound healing.

### 2.1 Types of wounds

Wounds embody a dynamic spectrum of tissue injuries, necessitating meticulous classification based on temporal, pathological, and etiological factors. Acute wounds arise suddenly, such as lacerations, abrasions, or contusions, triggering a well‐orchestrated healing cascade involving hemostasis, inflammation, cellular proliferation, and eventual tissue remodeling.^34^, ^35^ In contrast, chronic wounds, exemplified by diabetic ulcers, venous stasis ulcers, and pressure ulcers, present prolonged inflammation and compromised healing processes due to underlying pathologies like impaired vascular supply.^28^, ^36^, ^37^, ^38^ Surgical wounds stemming from planned interventions encompass categories such as clean, contaminated, and infected.^39^, ^40^ Each category of surgical wounds poses unique challenges in managing the delicate balance between facilitating healing and preventing infections.^41^, ^42^, ^43^ The classification of wounds plays a pivotal role in guiding tailored therapeutic interventions. This section provides a comprehensive overview of acute, chronic, and surgical wound classifications, setting the stage for a detailed exploration of traditional and modern treatment modalities. For a detailed summary of types of wounds, examples, and their characteristics, refer to Table 1.

**TABLE 1:** Classification of wounds and their characteristics.

	Wound type	Examples	Characteristics	References
1	Acute	Lacerations, abrasions, contusions	Sudden onset triggers a well‐orchestrated healing cascade involving hemostasis, inflammation, cellular proliferation, and tissue remodeling.	^1^, ^7^, ^34^, ^35^, ^44^
2	Chronic	Diabetic ulcers, venous stasis ulcers, pressure ulcers	Prolonged inflammation; compromised healing processes due to underlying pathologies like impaired vascular supply.	^28^, ^36^, ^37^, ^38^, ^45^
3	Surgical	Clean, contaminated, infected	Arising from planned interventions, each category poses unique challenges in balancing healing facilitation and infection prevention.	^41^, ^42^, ^43^

### 2.2 Traditional treatment methods

Wound care has traditionally relied on a spectrum of well‐established approaches to manage the complexities of tissue repair.^46^, ^47^ Primary closure, often suitable for acute wounds, involves surgically approximating wound edges to facilitate swift healing and minimize infection risks.^48^, ^49^, ^50^ Conversely, secondary intention healing allows wounds to progress through granulation tissue formation, wound contraction, and re‐epithelialization, mainly when primary closure is impractical.^1^, ^51^, ^52^ Applying topical antimicrobials, such as creams and dressings, is a common strategy to prevent or treat infections in acute and chronic wounds.^53^, ^54^, ^55^, ^56^, ^57^ Additionally, traditional wound dressings, including gauze and hydrocolloids, play a vital role in moisture management, infection prevention, and creating an optimal healing environment.^58^, ^59^, ^60^

However, challenges persist in current practices. Despite the application of antimicrobials, the risk of infection remains, especially in chronic wounds characterized by compromised immune responses.^61^, ^62^ Delayed healing is a common concern, often associated with persistent inflammation, impaired angiogenesis, and cellular dysfunction, necessitating extended treatment durations.^63^, ^64^ Furthermore, conventional methods may contribute to excessive scar formation, impacting the functional and esthetic aspects of wound healing.^65^ The limitations of existing treatments are multifaceted. Inadequate moisture control, a concern with traditional dressings, may lead to excessive dryness or prolonged dampness, hindering healing.^66^, ^67^ Biological inertia, the inability of standard treatments to address dynamic cellular and molecular alterations in chronic wounds, limits their efficacy in promoting timely and complete healing.^61^ Additionally, patient compliance plays a pivotal role in the success of traditional wound care, posing challenges when adherence to prescribed regimens is difficult to maintain.^68^ Table 2 below provides a detailed overview of these traditional treatment methods, delineating the types of injuries they are commonly used for and enumerating their respective advantages, disadvantages, and brief descriptions.

**TABLE 2:** Traditional treatment methods for wound healing.

	Treatment method	Brief description	Types of injuries	Advantages	Disadvantages	References
1	Primary closure	Surgical approximation of wound edges for swift healing suits acute wounds, but limitations exist for specific wound types.	Acute wounds	Promotes rapid healing Minimizes infection risk	Impractical for certain wounds Scar formation concerns	^48^, ^49^, ^50^, ^69^, ^70^, ^71^, ^72^
2	Secondary intention healing	Allowing wounds to heal naturally without closure is particularly beneficial for wounds where primary closure is challenging or impractical.	Wounds where primary closure is impractical	Facilitates healing without closure Suitable for irregularly shaped wounds	Prolonged healing time Increased risk of infection	^1^, ^51^, ^52^
3	Topical antimicrobials	Application of antimicrobial agents, available in various forms, to prevent or treat wound infections, applicable to both acute and chronic cases.	Acute and chronic wounds	Prevents or treats infections Versatile application options	Limited effectiveness against resistant strains Risk of hypersensitivity reactions	^55^, ^56^, ^57^, ^73^, ^74^
4	Advanced dressings	Utilization of specialized dressings, ranging from gauze to hydrocolloids, to manage moisture, prevent infections, and optimize the wound healing environment in various wound scenarios.	Different wound types, including chronic wounds	Supports moisture management Aids in infection prevention Creates optimal healing environment	Costs may be higher than basic dressings Efficacy depends on wound type and severity	^58^, ^59^, ^60^, ^75^, ^76^

### 2.3 Modern treatment methods based on nanotechnology

At the forefront of contemporary wound care strategies, nanotechnology emerges as a transformative force, harnessing the unique properties of nanomaterials to address inherent limitations in traditional therapeutic approaches. Nanomaterials, exemplified by carbon nanotubes, graphene, and nanoparticles, exhibit exceptional physicochemical attributes conducive to tailored therapeutic interventions. Their distinct surface characteristics and high surface area‐to‐volume ratio provide an intricate platform for precise interactions with biological systems, offering unparalleled opportunities to optimize wound healing processes.^18^, ^77^, ^78^, ^79^

A pivotal contribution of nanotechnology to wound care is the development of advanced drug delivery systems.^9^, ^80^ Nanostructured carriers, facilitating controlled and targeted release of therapeutic agents, present a paradigm shift in the pharmacological management of wounds.^81^, ^82^, ^83^ Such platforms enhance drug efficacy, mitigate systemic side effects, and overcome challenges associated with conventional drug administration.^84^, ^85^ In wound healing, a noteworthy study investigated the effectiveness of chitosan (CS)‐capped silver nanoparticles (Ch/AgNPs) for expediting the recovery of burn wounds. The comprehensive evaluations conducted in this study revealed that Ch/AgNPs exerted substantial anti‐inflammatory effects, facilitating increased re‐epithelialization and enhancing granulation tissue formation. These findings underscore the potential of Ch/AgNPs as a promising therapeutic agent, highlighting their ability to accelerate the intricate processes of wound healing and regeneration.^86^ Similarly, utilizing nanoliposomes loaded with the antibiotic vancomycin presents another compelling instance. This approach selectively targets bacteria in chronic wounds, demonstrating efficacy in treating infections while preserving the integrity of healthy tissues.^87^ Additionally, the advent of antimicrobial nanomaterials, exemplified by AgNPs and antimicrobial peptides (AMPs), introduces a formidable arsenal against bacterial infections in wounds, holding promise for both acute and chronic cases.^88^, ^89^, ^90^ An insightful investigation in the Journal of Materials Science & Technology demonstrated the remarkable efficacy of wound dressings incorporating AgNPs. This study revealed a significant reduction in bacterial colonization and a notable promotion of wound healing, mainly observed in diabetic foot ulcers.^91^ A pioneering strategy emerged by integrating a wound dressing featuring a blend of AMPs. This innovative therapeutic approach demonstrated noteworthy efficacy in effectively combating *Staphylococcus aureus* infections within diabetic foot ulcers, underscoring its potential as a promising and impactful intervention.^92^

**FIGURE 1:**
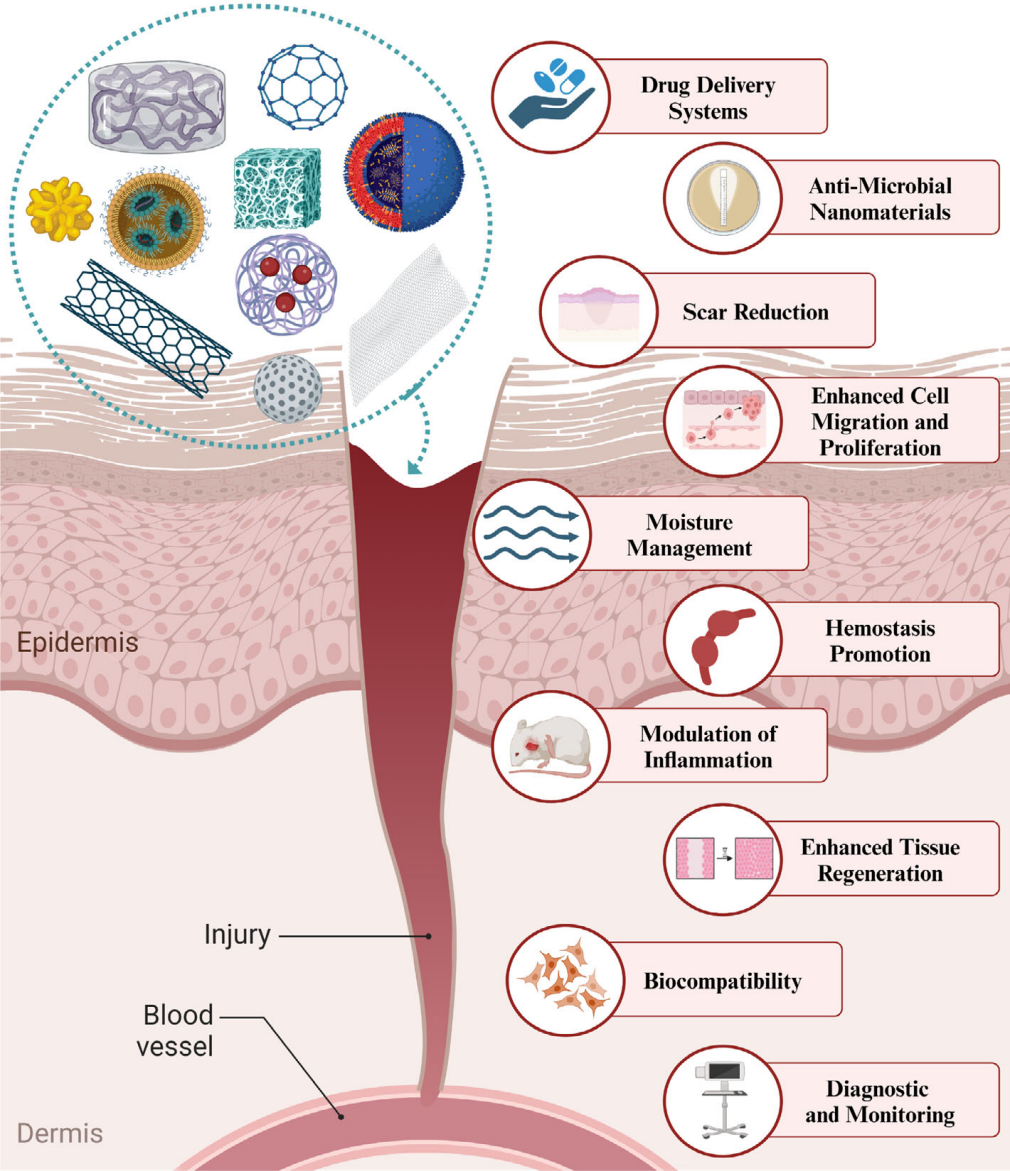
Impacts of nanomaterials on wound treatment and maintenance.

Nanotechnology extends its influence beyond pharmacological interventions, playing a crucial role in orchestrating enhanced tissue regeneration.^93^, ^94^ By modulating fundamental cellular processes such as adhesion, migration, and proliferation, nanomaterials craft an optimal microenvironment for tissue repair.^95^ An illustrative study demonstrated the effectiveness of liposomes loaded with vascular endothelial growth factor (VEGF) in fostering angiogenesis—new blood vessel formation—in diabetic foot ulcers.^96^ In a parallel vein, a separate investigation revealed the efficacy of nanozyme‐based therapy in treating liver fibrosis, a condition marked by the excessive formation of scar tissue.^97^ Furthermore, nanotechnological innovations contribute to developing advanced diagnostic and monitoring systems, utilizing nanoscale imaging techniques and smart nanomaterials to offer real‐time insights into wound status.^23^, ^98^, ^99^ Investigating wound healing, a study employs mRNA nanosensors (NanoFlare) topically applied to skin wounds, offering an innovative and real‐time approach for the targeted and semi‐quantitative examination of mRNA biomarkers in skin cells. This non‐disruptive method efficiently tracks and identifies wound healing stages in 2D and 3D in vitro and mouse models.^100^ Figure 1 presents a schematic delineating the impact and methodology of integrating nanotechnology in enhancing wound treatment modalities and their management.

As the integration of nanotechnology into wound care endeavors unfolds, it simultaneously presents unique challenges, necessitating a meticulous examination of biocompatibility, long‐term safety, and regulatory considerations.^10^, ^101^ In summary, the convergence of nanotechnology and wound care represents a dynamic scientific frontier with the potential to revolutionize contemporary clinical practices. The nuanced exploration of nanomaterial applications, spanning from drug delivery to tissue regeneration, highlights a transformative era wherein nanotechnology emerges as a critical innovator for enhancing wound healing strategies.

## 3 CARBON‐BASED NANOMATERIALS

Carbon nanomaterials are the most popular nanomaterials in the field of nanomedicine^27^ due to their unique properties, such as a high surface‐to‐volume ratio^102^ and biological and physicochemical properties in the field of tissue engineering, drug delivery,^27^ and imaging^102^ that have been considered. Carbon has many allotropes, such as carbon nanotubes, graphene, graphene oxide (GO), reduced GO, diamond nanoparticles, carbon nanofibers (CNFs), and nanocarbon dots.^103^ The antimicrobial activity of carbon nanomaterials is through the effect on the morphology and integrity of the cell membrane of bacteria, metabolic processes^102^ (depending on the severity of the damage^103^), biological isolation of bacteria from food, and causing oxidative stress oxidative in the presence or absence of light and the production of reactive oxygen species (ROS).^27^, ^104^ In addition to antibacterial properties, carbon nanomaterials have remarkable mechanical properties and the ability to induce wound healing, one of the characteristics of interest for wound dressings.^102^

In the following, the characteristics of carbon allotropes and their use in the production of wound dressings have been investigated.

To provide a clearer and more structured overview, Table 3 summarizes the major therapeutic mechanisms and biological effects of each type of carbon‐based nanomaterial employed in wound healing. This includes their antibacterial, anti‐inflammatory, angiogenic, antioxidant, and cell‐stimulating properties, along with proposed underlying mechanisms. Based on this summarized comparison, the following subsections further explore the specific characteristics and wound healing applications of each carbon allotrope in more detail.

**TABLE 3:** Therapeutic mechanisms and biological effects of carbon‐based nanomaterials used in wound healing applications.

Type of carbon nanomaterial	Antibacterial	Anti‐inflammatory	Angiogenesis	Antioxidant	Cell proliferation and migration	Mechanisms of action	References
Graphene‐based nanomaterials (e.g., GO, rGO)	✓✓	✓✓	✓	✓	✓✓	ROS generation, membrane disruption, protein adsorption, immunomodulation	^105^, ^106^
Carbon dots (CDs)	✓✓	✓	✓	✓✓	✓	Mimicking enzyme activity, ROS scavenging, and cellular uptake modulation	^107^
Carbon nanotubes (CNTs)	✓	✓	✓✓	✓	✓✓	Topographical stimulation, cell adhesion, growth factor binding	^108^
Carbon nanofibers (CNFs)	✓	✓	✓	✓	✓	Electroconductivity, ECM mimicry, scaffold for cell growth	^109^, ^110^
Nanodiamonds (NDs)	✓	✓✓	✓	‐	✓	Surface reactivity, protein immobilization, low toxicity	^111^
Fullerenes (C60)	✓✓	✓	‐	✓✓	✓	Antioxidant via ROS scavenging, mitochondrial protection	^112^
Carbon nano‐onions (CNOs)	✓	✓	✓	✓✓	✓	Multishell structure promotes sustained antioxidant effect, modulates inflammation	^113^, ^114^

*Note*: ✓✓ indicates strong and repeated evidence reported in multiple studies; ✓ indicates reported but limited evidence; “–” indicates the least or no reported evidence.

Abbreviations: ECM, extracellular matrix; rGO, reduced graphene oxide; ROS, reactive oxygen species.

### 3.1 Graphene‐based nanomaterials

Graphene is a two‐dimensional carbon nanomaterial^103^ that consists of a sp^2^ hybrid carbon network with a C‐C distance of 1.42 Å and an interlayer length of 3.4 Å.^27^ Graphene has electrical conductivity, high absorption of light, and excellent mechanical properties, which justifies its use in various industries such as the steel industry, energy harvesting, and strain sensors.^115^

In addition to the remarkable features of graphene, such as amphiphilicity, high surface performance, biocompatibility, and ease of drug loading,^116^ this two‐dimensional nanomaterial has been widely used in medical industries such as coating application,^117^ bioimaging,^118^ phototherapy,^119^ drug delivery,^120^ and electroconductive scaffolds such as bone,^121^ nerves,^122^, ^123^, ^124^ and heart.^125^, ^126^

Still, due to their high surface energy, graphene nanoparticles tend to agglomerate, making their use in biomaterials challenging.^127^ By activating covalency and synthesizing GO or reduced graphene oxide (rGO), which contain hydroxyl, epoxy, and carboxyl groups, the amount of dispersion^127^ and their solubility in water can be improved, thus preventing agglomeration.^103^ Graphene via chemical vapor deposition,^128^ GO with methods such as Brodie and Hummer techniques,^129^ and microbial reduction methods.^27^, ^103^, ^130^

In the oxidation of graphene, planar sp^2^ hybridization carbons are transformed into tetrahedral sp^3^ hybridization under covalent functionalization, which is why the conductivity of GO decreases compared to graphene.^127^ Due to oxygenated functional groups, GO can be bonded with carbodiimide, isocyanates, and divinyl sulfone groups to connect other compounds.^131^ GO has a suitable dispersion in water and a high cross‐sectional area, which activates the possibility of angiogenesis by phosphorylating active oxygen and nitrogen species.^102^ Depending on the oxidation degree and thickness, GO has tunable mechanical properties. Young's modulus and intrinsic strength of GO monolayers are 150 ± 250 and 50–30 GPa, respectively.^132^

According to the ability of carbon nanomaterials to inhibit the growth of bacteria, research has shown that graphene oxide nanowalls (GONWs), reduced graphene oxide nanowalls (RGNWs), GO, and rGO can destroy the bacterial membrane like a sharp knife or by producing ROS and lipid extraction, leading to stopping their growth.^133^, ^134^

**FIGURE 2:**
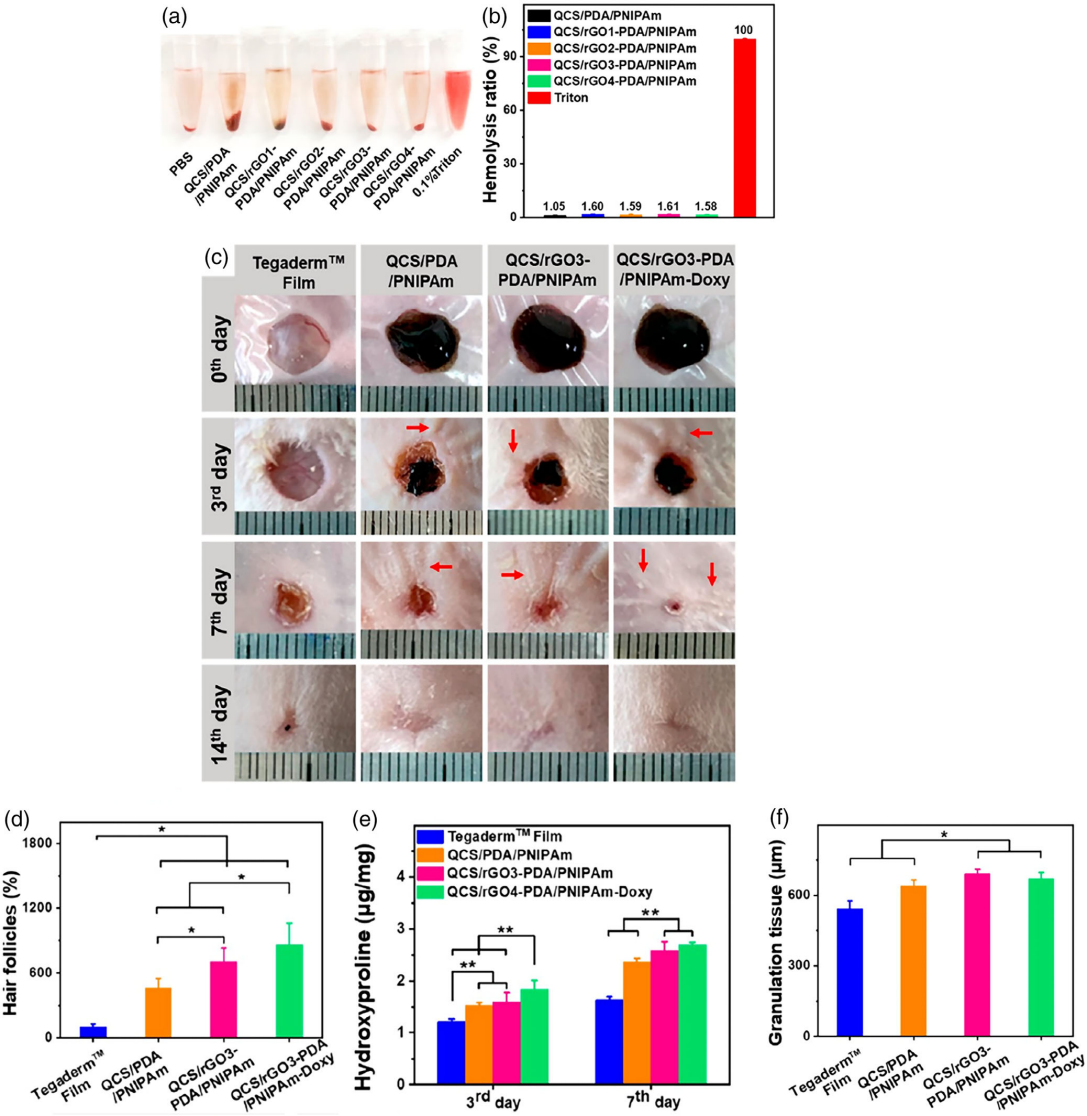
(a) Representative image of the hemolysis experiment. (b) In vitro evaluation of the hemolytic activity of all hydrogels. (c) Photos of the wound area on Day 3, Day 7, and Day 14 for Tegaderm film dressing (control), quaternary chitosan/polydopamine/poly *N*‐isopropioacrylamide (QCS/PDA/PNIPAm), QCS/rGO3‐PDA/PNIPAm, and QCS/rGO3‐PDA/PNIPAm‐Doxy, and traces of wound bed closure for 14 days with each treatment. (Red arrows) Traces of skin contraction caused by hydrogel‐assisted wound closure. (d) The number of hair follicles in all groups on Day 14, and the number in the control group was set to 100%. (e) Amount of collagen in all groups by determining hydroxyproline. (f) Statistical granulation of tissue thickness for different treatments on Day 7.*p < 0.05, **p < 0.01.^135^

Li et al.^135^ designed multifunctional quaternary chitosan (QCS), rGO coated with polydopamine (PDA) (rGO‐PDA) and poly(*N*‐isopropioacrylamide) (PNIPAm). They observed that, in addition to the antibacterial properties of CS, rGO, and PDA, which have good photothermal properties, strengthen the antibacterial property with near‐infrared radiation (NIR) radiation and increase the temperature. They found that the QCS/rGO‐PDA/PNIPAm wound dressing ultimately killed all *Escherichia coli* and *S. aureus* bacteria after 10 min of NIR irradiation. In the blood test results (Figure 2a,b), the hemolysis compatibility of all hydrogels was less than 2%, and the percentage of viable mouse fibroblast cells (L929) was between 82% and 93%. In a quantitative evaluation of the expression of interleukin 6 (IL‐6) and cluster of differentiation 31 (CD31) after 7 days, which are inflammatory cytokines and angiogenic proteins, respectively, they observed that the level of IL‐6 expression in the QCS/rGO3‐PDA/PNIPAm wound dressing containing doxycycline hydrochloride decreased and reached a constant level, which indicates that the level of inflammation has decreased and the immune response has been controlled. The expression of CD31 also increased on Day 7 in the QCS/rGO3‐PDA/PNIPAm‐Doxy sample, indicating that the vascularization process increased after the reduction of immune responses and inflammation in the presence of rGO and doxycycline hydrochloride.

In fact, according to Figure 2c, the wound volume of the QCS/rGO3‐polyvinyl alcohol (PVA)/PNIPAm and QCS/rGO3‐PDA/PNIPAm‐Doxy samples decreased on Day 7 and shrank by 83.6% and more than 90% of the wound volume, respectively. This indicates better wound healing in the presence of rGO and doxycycline hydrochloride. Hair follicle formation by Day 14, collagen production, and tissue granularity thickness by Day 7 (Figure 2d–f) in QCS/rGO3‐PVA/PNIPAm and QCS/rGO3‐PDA/PNIPAm‐Doxy samples increased compared to samples without rGO and control, indicating an increase in the speed of wound healing. In general, after wound formation, in the QCS/rGO3‐PDA/PNIPAm‐Doxy sample, the level of inflammation was high until the third day, which was reduced by the presence of rGO along with doxycycline hydrochloride by the seventh day, and new tissue formation was started, and after 14 days, complete healing occurred. This means the electrical conductivity of rGO and the anti‐inflammation and anti‐microbial effect of both doxycycline hydrochloride and rGO closed the wound after 14 days.

In the study of Fernández et al.,^136^ rGO‐nanofibril cellulose (nFC)–tannin (TA) and PDA composite were made to be used as a wound dressing. As a result of cytotoxicity, the percentage of human fibroblast cells in the rGO/nFC/TA sample was 80%. It was reported that in the in vitro wound healing test (scratch), the migration of human fibroblast cells to the wounded area was observed after 48 h, which indicated the presence of rGO accelerates the cell migration, the repair process of the wound, and ultimately causes wound closure.

**FIGURE 3:**
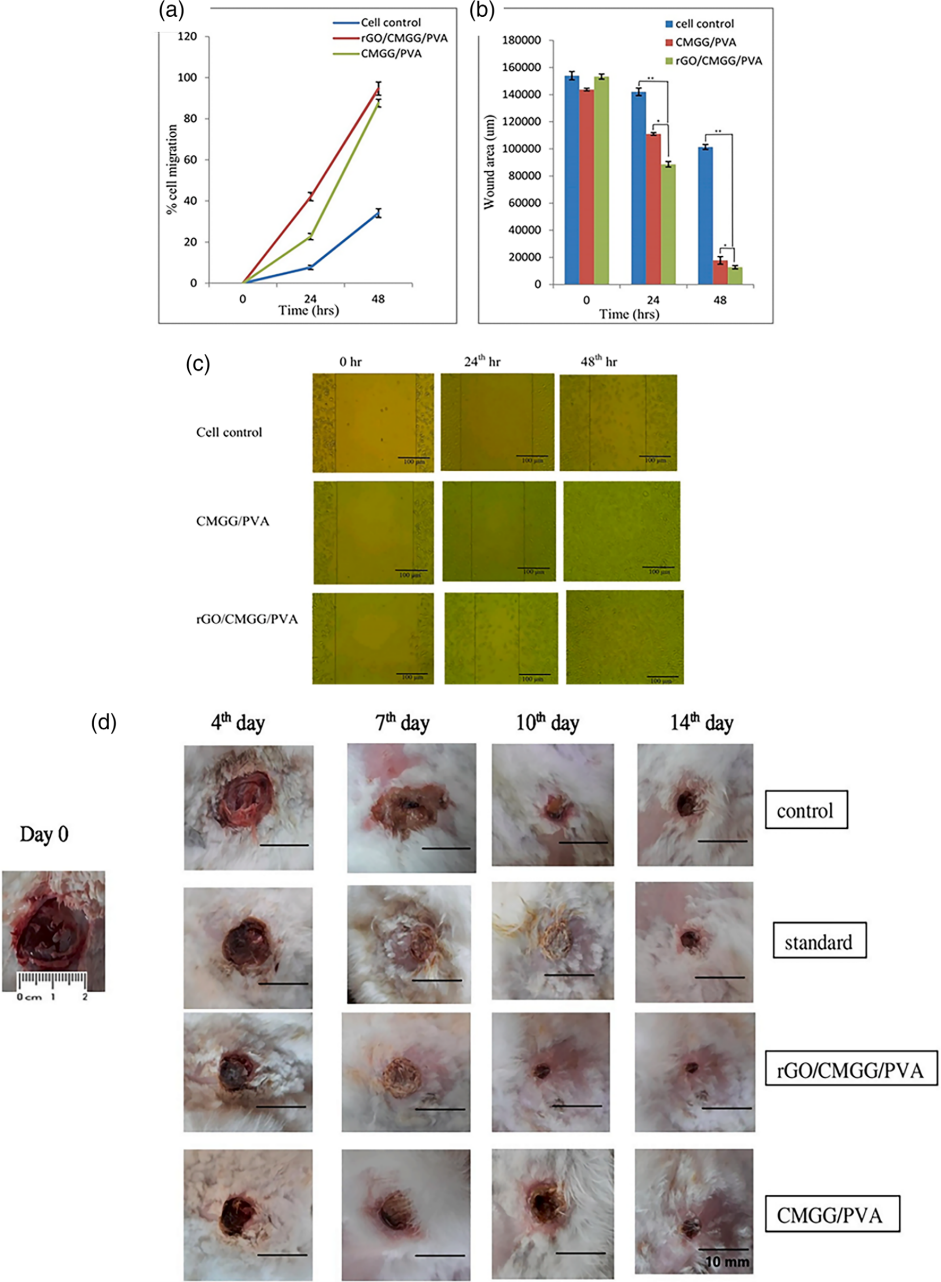
Results of the in vitro scratch assay study: (a) = percent cell migration of the 3T3‐L1 cell line; (b)= representation of the wound closure area, * indicates significance and ** indicates high significance (*p* < 0.05); (c)= inverted microscopic images of the migration of fibroblast cells toward the scratch (magnification: 10×). (d) = digital images showing wound areas in rabbits (scale bar = 10 mm).^137^ CMGG, carboxymethyl guar gum; PVA, polyvinyl alcohol; rGO, reduced graphene oxide.

Another study showed that carboxymethyl guar gum (CMGG) (rGO) and PVA nanofiber scaffolds have high porosity and proper moisture retention. It was reported that the percentage of hemolysis in the sample containing rGO was 3.37% and in the control sample, 1.71%, which has also shown to be hemocompatible with permissible hemolysis ranges. The percentage of migration of fibroblast cells to the wound site during 48 h was significantly higher in rGO/CMGG/PVA compared to CMGG/PVA. The wound area after 48 h had also become much less, which was considered to be due to the faster migration of fibroblasts due to the presence of rGO, which stimulates cellular activity with its conductive properties. Also, in the animal test results, wound closure after 14 days in the sample containing rGO (rGO/CMGG/PVA) was similar to the standard group (treatment with povidone iodine). After the fourth and seventh days, the wound sizes of the (CMGG/PVA) groups and (rGO/CMGG/PVA) reported less than the control group, which was attributed to the induction of cell division, proper swelling, and moisture retention by rGO and CMGG (Figure 3a–d).^137^

In another study, by making an asymmetric scaffold sensitive to NIR containing a layer of CS‐rGO hydrogel and an electrospun membrane of PCL_cellulose acetate (CA), they investigated its application in skin tissue. They observed that increasing the amount of rGO increases the temperature of the scaffold under NIR irradiation, that is, after 10 min, the temperature increases by 9.7 ± 0.7 and 12.4 ± 0.3°C in the CS_rGO100 and CS_rGO200 samples, respectively, and can produce a mild hyperthermic effect that helps in wound healing without damaging the cells. Cell studies also observed that the percentage of living NHDF cells increased in both CS_rGO100 and CS_rGO200 samples, and cell proliferation (superior to 70%, after 1 and 3 days) and adhesion occurred against sequential NIR radiation (with a power of 1.7 W/cm^2^ and a duration of 10 min). In the antibacterial test, after three cycles of NIR irradiation to the samples, the percentage of *E. coli* decreased significantly from 84 to 16 in CS_rGO100, from 88 to 8 in CS_rGO200, and the percentage of *Pseudomonas aeruginosa* also decreased significantly from 90 to 12 in CS_rGO100, from 88 to 6 in CS_rGO200. These data validate the safety of the mild hyperthermia treatment.^138^

**FIGURE 4:**
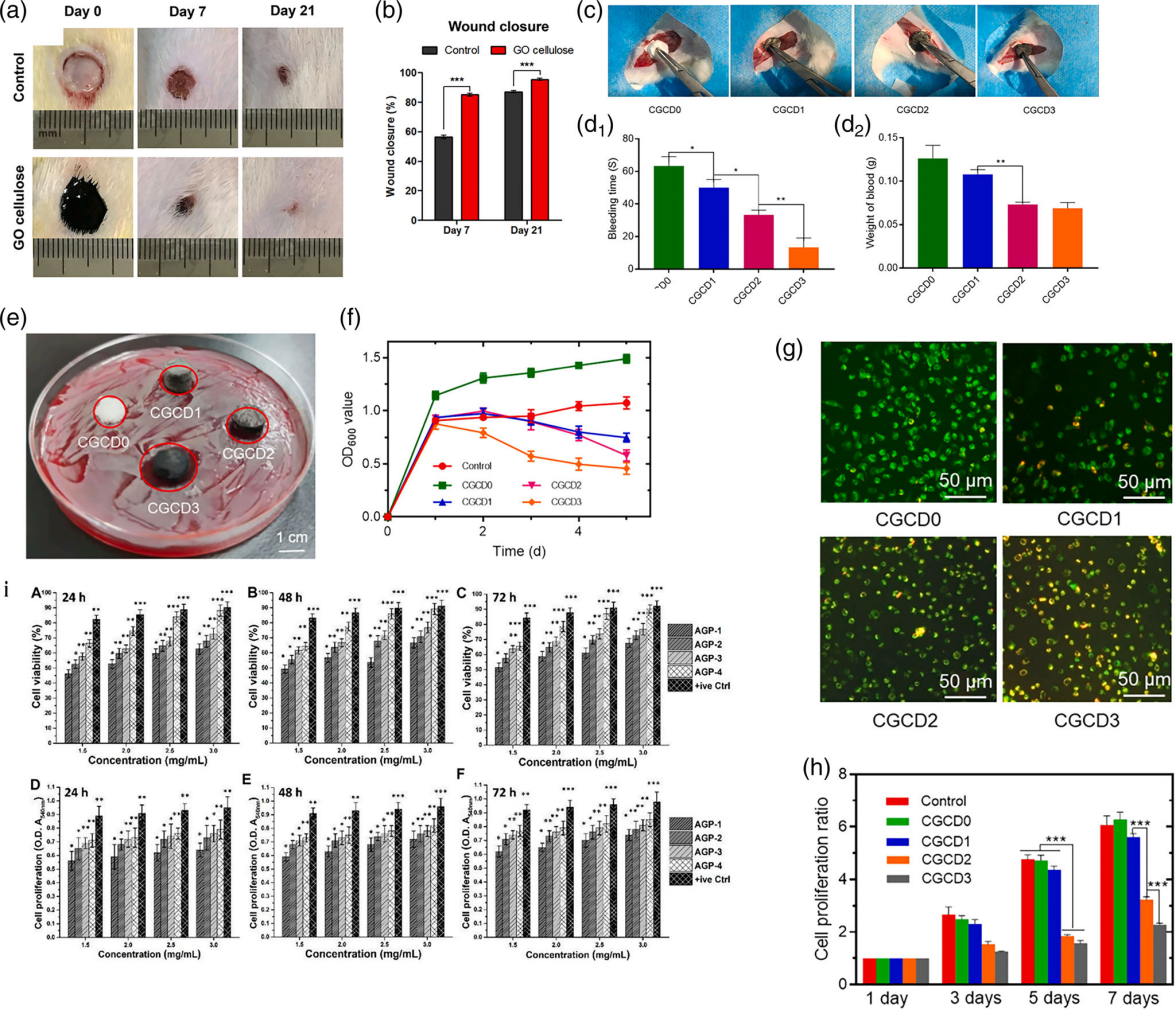
Complete evaluation of skin wound in rats treated with or without graphene oxide (GO)‐cellulose nanocomposite. (a) Photos of skin wounds of the control or GO‐cellulose nanocomposite‐treated groups on Days 0, 7, and 21 after wound induction. Ruler units are mm. (b) Percentage of wound closure was measured using ImageJ. The results were compared with the control group. Differences were assessed using one‐way ANOVA. ***p < 0.0001.^139^ (c) Photographs of dressings to control bleeding in each group. (d_1_) Hemostasis time of the foam gel dressings in each group. (d_2_) Blood loss weight of the foam gel dressings in each group *p < 0.05, **p < 0.001. (e) Photographs of the antibacterial zone of the foam gel dressings against *Staphylococcus aureus* in each group. (f) The statistical graph of antibacterial OD value of foam gel dressings against *S. aureus* in each group. (g) Immunofluorescence image of LIVE/DEAD staining of 3 T3 fibroblasts cultured in PBS extract of a foam gel dressing for 24 h. (h) Experimental diagram of proliferation of 3 T3 fibroblasts cultured in foam gel dressing extract within 7 days ***p < 0.0001.^140^ (i) Biocompatibility tests were carried out with different concentrations of composite hydrogels to determine the bioactive behavior. Biocompatible behavior was examined by cell viability (A–C) and cell proliferation (D–F) using osteoblastic cell line (MC3T3‐E1). Gelatin (0.1%) serves as a positive control. *p < 0.05, **p < 0.001, and ***p < 0.0001.^141^

Soliman et al.^139^ investigated the effect of GO‐cellulose nanocomposite on wound healing. The percentage of viable immortalized human vascular endothelial cells (EA.hy926) endothelial cells was increased by Day 7 (~ 110%). In the in vitro wound scratch test, they observed that GO‐cellulose nanocomposite increases the migration of cells to the scratch site for 3 days, and the scratch site closes, which indicates the stimulation of cell migration and cell activity in the presence of GO. In the in vivo studies, they also found that GO‐cellulose nanocomposite wound dressing significantly closed the wound in mice after 21 days compared to the control sample. The edges of the wound were pulled together and closed prematurely, to the point that traces of skin structures, such as hair follicles and subcutaneous glands, reappeared (Figure 4a,b).

Specifically, Soliman et al. observed the stages of wound healing in in vivo studies, which initially, due to the presence of a wound, the inflammatory phase of the wound was established by the presence of neutrophils and macrophages, which are responsible for cleaning the wound and preparing the environment for healing. With the reduction of inflammatory cells, migration of fibroblasts and endothelial cells and the initiation of new blood vessels were observed. This was followed by collagen deposition, the reappearance of skin appendages such as hair follicles and sebaceous glands. Also, in addition to the moisture retention ability of cellulose, GO can eliminate ROS and regulate inflammation to accelerate wound regeneration. Therefore, this scaffold has shown good potential for skin purposes.

Xie et al.^140^ fabricated an oligosaccharide wound dressing on a calcium alginate foam substrate modified with GO. They observed that the presence of GO, due to its tendency to bind to platelets, stopped bleeding and created hemostasis. The blood clotting time decreased from 65 s in the sample without GO to 10 s in the sample containing 3% GO. In the biocompatibility test, the fibroblast cells had a spindle‐shaped morphology, and the scaffold showed no cytotoxicity. Still, the percentage of cell division decreased with the increase of GO amounts. This article shows that the cellular compatibility of GO is strongly dependent on the concentration. It was also observed that the inhibition zone of *P. aeruginosa* was a function of GO concentration, and by increasing the amount of GO, the density of bacteria will decrease, thereby preventing infection and increasing the level of wound healing (Figure 4c–h).

Khan et al.^141^ examining the properties of PVA‐GO hydrogel modified with arabinoxylene, along with observing the antibacterial property due to the sharp edges of GO, also proved their blood compatibility property and observed that less than 1% of hemolysis occurred and suggested that this amount of hemolysis can be due to the sharp edges of GO. They found that the percentage of osteoblastic cell line (MC3T3‐E1) cells increases with increased concentration of hydrogels in contact with the cells. Factors such as the electrical conductivity of GO, the high surface ratio of GO, and the oxygen functional groups in GO that tend to form hydrogen bonds with the cells provide conditions for cell adhesion and survival (Figure 4i).

In addition to the suitable properties of GO and rGO nanoparticles mentioned in the previous content, we can say their amphiphilic property has solved the hydrophobicity of graphene.^131^ Another advantage is the existence of hydrophilic groups in the structure of GO, which allows for the creation of covalent and non‐covalent bonds with biomolecules. It should also be considered that graphene nanosheets with a size of less than 100 nm can cause cytotoxicity, inflammation, and genotoxicity.^142^ Functionalized graphene containing oxygen can quickly enter the cell and cause functional disorders in the cell membrane.^143^ As a result, in addition to mechanical and electrical properties and supporting cellular behavior, the size of graphene particles should also be considered in biomedical applications, such as wound dressings.

### 3.2 Carbon‐based dots

Carbon‐based dots (CDs), including graphene quantum dots (GQDs) with a size less than 20 nm, carbon nanodot (CNDs), and carbon quantum dots (CQDs)^144^ with a size less than 10 nm due to their chemical stability, fluorescence property, good conductivity, optical stability against blinking and photobleaching phenomena, and biocompatibility have attracted the attention of researchers in multifunctional drug‐carrier systems, bioimaging, and tissue engineering.^145^, ^146^ These carbon nanoparticles are produced by top‐down methods, such as reducing the size of materials from the macro scale (graphene sheets or carbon nanotubes) to carbon nanoparticles or bottom‐up processes, such as polymerization or carbonization.^147^ Due to features such as a lower price than AgNPs and metal oxides, preparation from renewable sources, apoptosis of bacteria by causing physical damage, and oxidative stress, they are suitable for wound dressings.^145^ Also, in cell studies, these materials have shown less toxicity and better biocompatibility than carbon nanotubes (CNTs) for macrophage cell line (Raw264.7), NIH3T3, and fibroblast cells. Methods such as injecting CDs into the tail vein of vertebrates and making hydrogel and nanofiber scaffolds can be used to treat wounds.^145^ CDs can be functionalized with hydroxyl, carboxyl, carbonyl, amino, and epoxy groups, which cause adjustment of hydrophilicity and hydrophobicity, and thermodynamic stability in solvents, especially water.^148^ Hydrothermal solution heating, microwave, and microplasma synthesis methods synthesize CQD blue, green, yellow, and red CQDs.^149^

**FIGURE 5:**
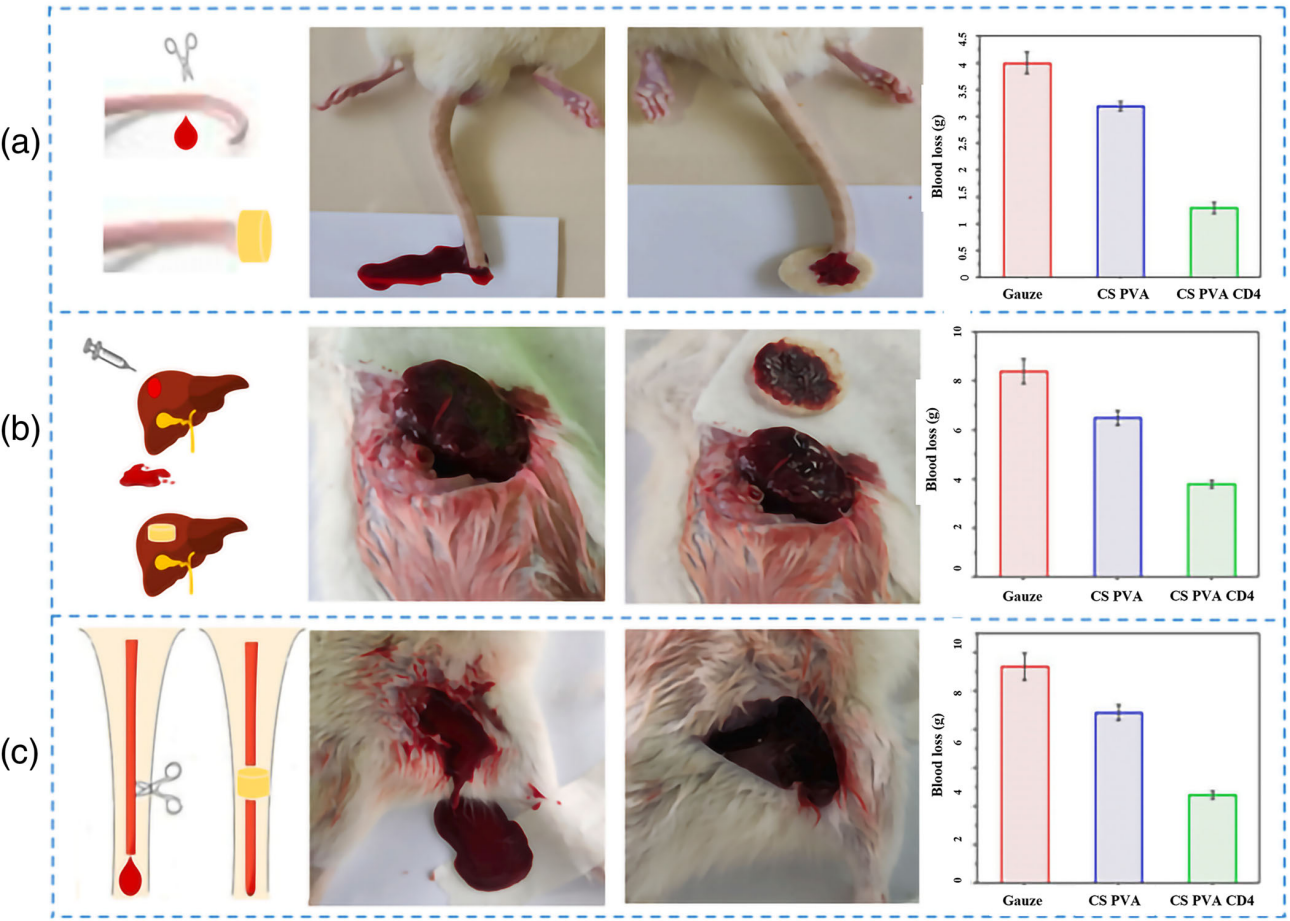
Approximation of cluster of differentiation 4 effect in (a) rat tail amputation, (b) rat liver injury, and (c) rat leg artery injury models.^150^ CS, chitosan; PVA, polyvinyl alcohol.

Shakiba‐Marani et al.^150^ By making CS/PVA/CD nanocomposite sponges, the effect of CDs on mechanical properties, hydrophilicity, and cellular and animal behavior was studied. They observed that the addition of CDs increased its hydrophilicity, and the flexibility of the scaffold also increased with a reduction in yield strength. In the whole blood clotting test, CS/PVA/CD nanocomposite sponges showed the lowest Blood Clotting Index. Also, in animal tests, it showed hemostatic effects because CDs reduced blood loss and rapid hemostasis due to their ability to form complexes with blood proteins and iron ions in blood plasma. As can be seen in the figure, the amount of blood loss in the tail, liver, and foot of the mouse in the gauze sample is almost double that of the CS/PVA/CD sample. Blood cells and platelets are necessary for the faster formation of a thrombus, and the combination of these factors accelerates the coagulation process. No toxicity was shown for L929 cells, with the results reporting a percentage of live cells above 70% (Figure 5).

Wang et al.^151^ implanted the composite film of PVA‐CQDs doped with lanthanum (La@N‐P‐CQDs) in mice and observed that the cell growth rate was above 95% and the hemolysis rate was below 5%, and this composite maintained the morphology of red blood cells. According to the fluorescence images of human lung cancer cells incubated with La@N‐P‐CQDs, these nanoparticles effectively entered the cytoplasm and nucleins without harming cell structure. These results support the necessity of their use in the biomedical field. In an in vitro antibacterial test, the anionic bacterial membrane was attracted to the positive membrane of these nanoparticles, and by increasing the concentration of nanoparticles, the antibacterial effect increased.

In in vivo experiments, after wound formation and *S. aureus* treatment by Day 2, the bacterial infection increased, and the scaffold treatment started. In the first 3 days of scaffold treatment, the migration of inflammatory cells to the wound site increased; however, the PVA/La@N‐P‐CQDs showed fewer inflammatory cells and more fibroblast cell migration. After the bacteria were killed and the area was prepared for healing, the formation of an epithelial layer began by Day 7, along with the growth of blood vessels and hair follicles in the PVA/La@N‐P‐CQDs group. Finally, by the end of Day 14, this group contained the thickest epidermis, the most active fibroblasts, and the most blood vessels and hair follicles, and wound closure had occurred more frequently.

Zmejkoski et al.^152^ prepared bacterial cellulose‐GQDs composite hydrogels. They also observed the inhibitory and bactericidal effects of the composites, especially in bacterial cellulose‐2 mg GQDs. In cell viability tests, the cell growth rates were above 95%, and in the scratch test results, they observed that the hydrogels were biocompatible and stimulated the migration of fibroblast cells. Also, in the bacterial cellulose‐2 mg GQDs sample, the capacity for wound fluid absorption after 48 h was ~120%, which helps maintain wound moisture and accelerate tissue repair. In addition, the gene expression of influential factors in angiogenesis, such as endothelial nitric oxide synthase (eNOS) and vascular endothelial growth factor A (VEGFA), endothelial factors, and matrix metalloproteinase‐9 (MMP 9), which contribute to wound healing, had occurred.

In the study of Moniruzzaman et al.^153^ green and yellow CQDs (G‐CQDs and Y‐CQDs) were used as a factor to prevent the activity of enzymes such as collagenase, tyrosinase, and elastase, which reduce skin elasticity against factors such as temperature, UV radiation, humidity, and stress. They observed a higher antioxidant activity of 87.1 ± 3.6% at a concentration of 100 μg/mL in the Y‐CQD‐GelMa hydrogel, which prevents the production of free radicals, and found that with increased antioxidant activity, less ROS is produced under stress conditions (UV radiation and H_2_O_2_); therefore, the expression of aging‐related enzymes (collagenase, tyrosinase, and elastase) is inhibited, significantly increasing cell survival. Also, along with antibacterial properties, GelMA‐CQD composite hydrogels showed good biocompatibility for human dermal fibroblast cells (hDFs) due to their antioxidant and antiaging properties. In animal studies, after implantation of GelMA‐CQD scaffolds at the wound site, in the first 7 days, the infiltration of skin cells, macrophages, and endothelial cells occurred first, and after the wound site was cleared and inflammation was controlled, the growth of the epidermal layer and the formation of connective tissue and the activation of fibroblasts for collagen secretion occurred by the end of the seventh day. In the last 7 days of the animal test, skin formation with re‐epithelialization and the formation of new blood vessels and an increase in epidermal ridges and hair follicles were observed, and the wound closure rate was ~85%.

According to what was mentioned, compared to other carbon materials, the research on CDs is in the early stages. Until now, there has been no systematic and scalable synthetic method. Therefore, the factors involved in the synthesis, such as temperature, time, and pH, should be investigated to solve this challenge. In addition, cell and animal toxicity studies should be addressed more widely.

### 3.3 Carbon nanotubes

CNTs are one‐dimensional nanomaterials of other carbon allotropes. Carbon nanotubes are placed together as tubular graphene sheets, creating a cylindrical structure. These materials are divided into three groups: single‐walled carbon nanotubes (SWCNTs), double‐walled carbon nanotubes (DWCNTs), and multi‐walled carbon nanotubes (MWCNTs). The lengths of SWCNTs and MWCNTs are 100–1000 and 15,000 nm, respectively, and the diameters are 1–2 and 100–5 nm, respectively.^154^ In MWCNTs, the distance between each wall is about 3.4 Å, which is synthesized with the help of methods such as heterogeneous decomposition of carbon monoxide and chemical vapor deposition.^155^, ^156^, ^157^ In addition to the mechanical and electrical properties due to having properties such as antibacterial, angiogenesis, and promoting the migration and adhesion of fibroblastic cells, they are used in wound dressings^158^ and they are used as an agent to increase the thickness of the epithelium layer.^159^

The bacteriostatic attributes of CNTs are attributable to the impairment of the cell membrane in microorganisms through direct interaction. This interaction leads to the oxidation of the membrane, and the generation of ROS can inflict harm upon the biological molecules of bacteria. Furthermore, it may indirectly induce DNA destruction.^160^, ^161^

**FIGURE 6:**
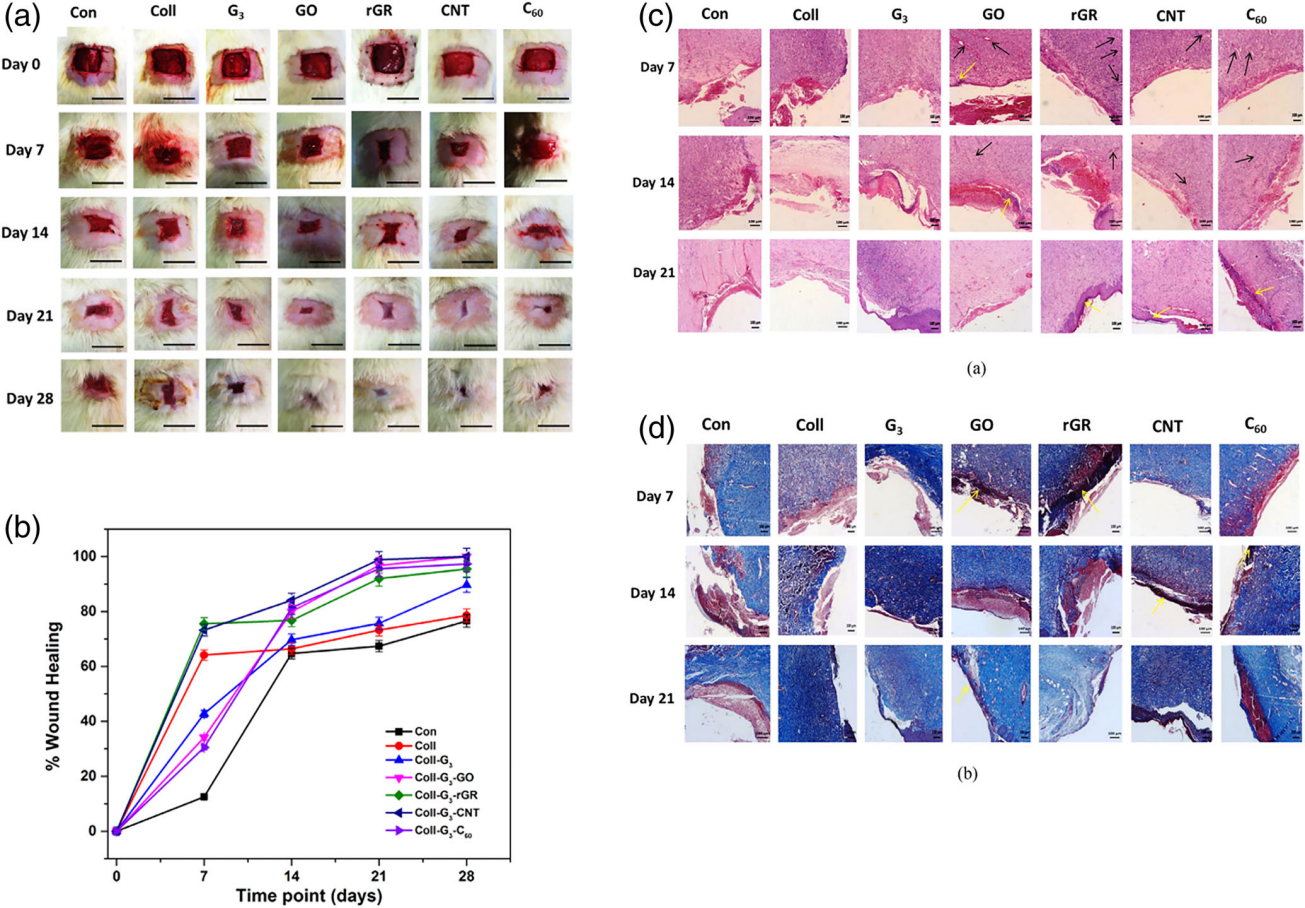
Representative photos of (a) the healing process of full‐thickness excision wounds, (b) percentage wound closure of the area on Days 7, 14, 21, and 28 after treatment with different collagen‐based scaffolds. Scale bar: 2 cm. Images of (c) H&E (d) Masson Trichrome stain sections of 7, 14 and 21 days of wounded tissue after treatment with another form of collagen scaffolds and the results are compared to the control group (scale bar 10 μm). Black arrows indicate blood vessel formation and yellow arrows indicate the interface between scaffold and tissue [Con: control, Coll: collagen, G3: triethoxysilane poly(amidoamine) dendrimer generation 3 [TES‐PAMAM‐G3 or G3], graphene oxide [GO], **reduced graphene [rGR]**, and fullerene [C_60_]).^162^

To investigate the effects of improving the biocompatibility and mechanical properties of carbon nanomaterials, Vedhanayagam et al.^162^ made a scaffold of triethoxysilane poly(amidoamine) dendrimer generation 3 (TES‐PAMAM‐G3)‐collagen‐carbon nanomaterials. Among the scaffolds functionalized with GO, rGO, and fullerene, the MWCNT‐TES‐PAMAM‐G3 collagen scaffold was a promising choice for wound healing. With the addition of carbon nanomaterials, the nature of the scaffolds changed from the elastomeric state (due to the presence of collagen with elastic properties) to the plastic state, and the mechanical properties improved. This result was attributed to the parallelism of MWCNT with carbon fibers. Due to the proper interaction between MWCNTs with collagen and dendrimer, slow biodegradation took place, which affected the cell results and prevented stress on the cells. Thus, the percentage of L929 (Mouse fibroblast) cells was higher in the sample containing MWCNTs, although the percentage of cells decreased linearly with the increase of carbon nanomaterials. In animal studies, they observed that TES‐PAMAM‐G3 collagen scaffolds containing carbon nanomaterials such as MWCNT, GO, **reduced graphene (rGR)**, and fullerene (C 60) showed a significant reduction in wound area at Day 7. At Day 14, the wound area in all groups showed a significant reduction in the MWCNT‐treated group compared to the other groups. Then, at Day 21, healing was faster in the MWCNT‐TES‐PAMAM‐G3 treated groups because the wound was covered by an epithelial layer. While in the control groups, collagen and TES‐PAMAM‐G3 were not yet fully formed. Also, at Day 28, hair follicles were observed in the MWCNT‐treated wounds. These findings indicate that collagen scaffolds attached to carbon nanomaterials could serve as a better substrate for use in wound healing. They were accompanied by faster epithelialization and greater collagen formation, and as a result, healing occurred more quickly during treatment. In the histopathology results on the seventh day, a large number of inflammatory cells were observed in the control group compared to the groups containing carbon nanomaterials. The images indicated the presence of newly formed blood vessels and granulation tissue, and new epithelial layers in the groups containing carbon nanomaterials. From the seventh day onwards, the thickness of the newly formed epidermis in the carbon nanoparticle‐mediated scaffold became thicker than in the other groups. H&E images after 21 days showed the presence of dense fibroblasts and keratinocytes and abundant collagen and mature hair follicles in the wound treated with carbon nanoparticle scaffolds. This was attributed to the migration of fibroblasts and keratinocytes to the wound site. (Figure 6a–d).

**FIGURE 7:**
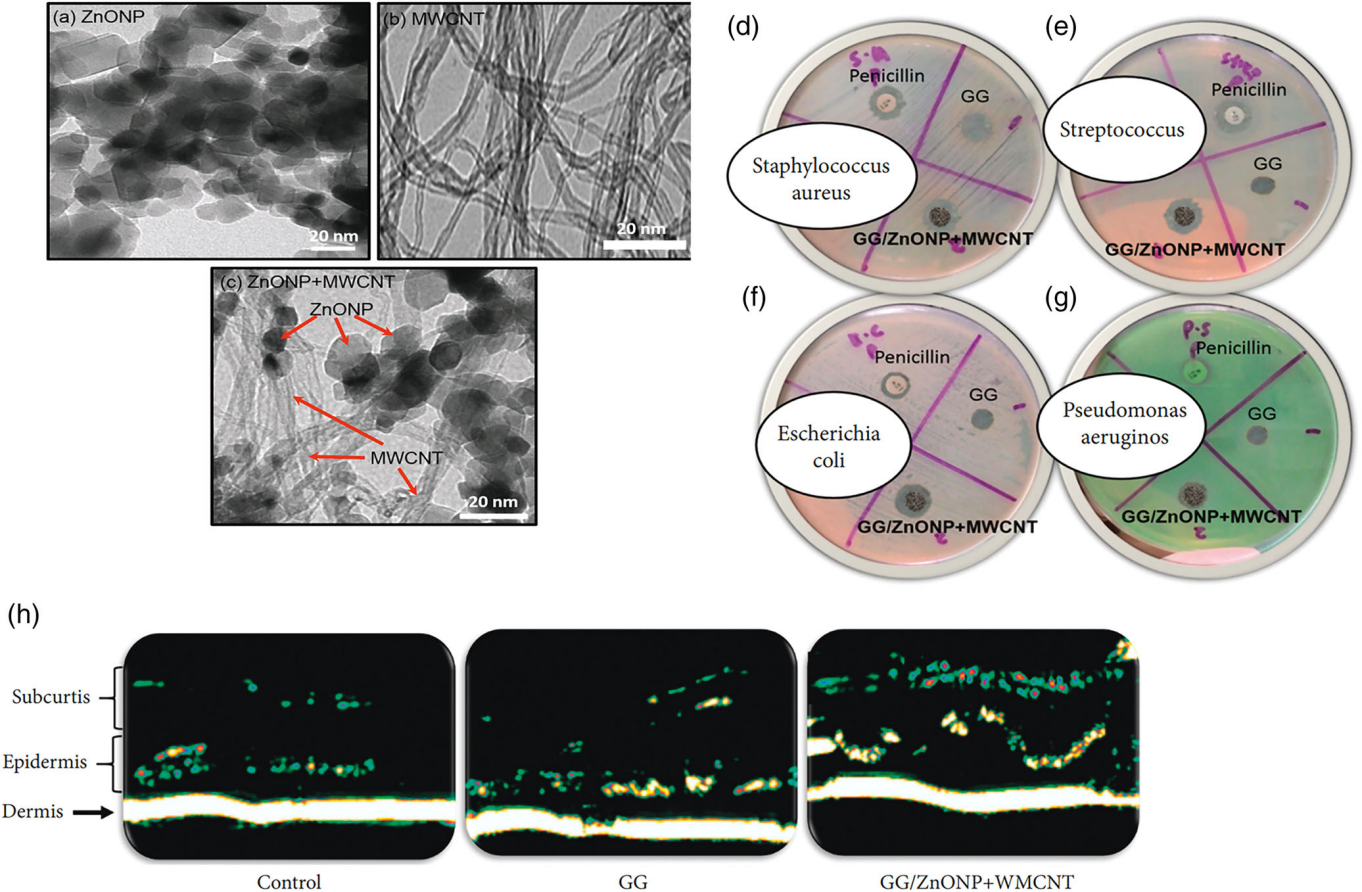
TEM images of (a) zinc oxide nanoparticles (ZnONP), (b) multi‐walled carbon nanotubes (MWCNT), and (c) ZnONP + MWCNT nanocomposites. Results of agar well diffusion test of control samples (penicillin), pure Gellan Gam (GG), and GG/ZnONP + MWCNT bio nanocomposite films against (d) *Staphylococcus aureus*, (e) *Streptococcus*, (f) *Escherichia coli*, and (g) *Pseudomonas aeruginosa* bacteria. (h) Ultrasound photos of the healed wound area in Sprague–Dawley rats treated with control GG, GG/ZnONP + MWCNT on Day 14.^163^

In the study of Liu et al.,^163^ by making Gellan Gam (GG) nanocomposite film containing zinc oxide nanoparticles (ZnONP) and MWCNT, they found that by adding ZnONP and MWCNT, the film became spongy, suitable for absorbing body fluids and treating wounds. ZnONP and MWCNT increased the scaffold's antibacterial activity against *S. aureus*, *Streptococcus*, *E. coli*, and *P. aeruginosa* compared to the GG sample. After 14 days, the wound contraction rate in the MWCNT‐containing sample was almost 100% because the formation of granulation tissue, new blood vessels, and epithelial tissue had occurred, while in the non‐MWCNT and non‐ZnONP samples, 77.40% and 90.76% of healing had occurred. In the ultrasonic images taken on the 14th day of animal studies, they saw the formation of thicker and more transparent layers of the dermis, epidermis, and subcutis in the GG/ZnONP + MWCNT sample than in the control, and this was attributed to the expression of collagen, porous morphology of the scaffold for passaging body fluids, as well as the antibacterial properties and stimulation of angiogenesis of ZnONP and MWCNT (Figure 7a–h).

Chen et al.^164^ fabricated carboxylated CS/CNT nanoparticles with a diameter of 150–250 nm to control the slow release of isoniazid for treating surgical wounds in bone tuberculosis. Carbon nanotubes can cross physiological barriers to deal with *Mycobacterium tuberculosis* in the lungs. In the results of immunohistochemistry, they observed that the number of CD3+ and CD4+ T lymphocytes decreased significantly, indicating the destruction of *M. tuberculosis* in the presence of the isoniazid drug and carboxylated CS/CNT, as well as a reduction in the area of involved by *M. tuberculosis*. So the wounds healed after 21 days due to reduced immune responses (CD3+ and CD4+ T lymphocytes) and infection caused by *M. tuberculosis* due to the presence of CS and CNT.

**FIGURE 8:**
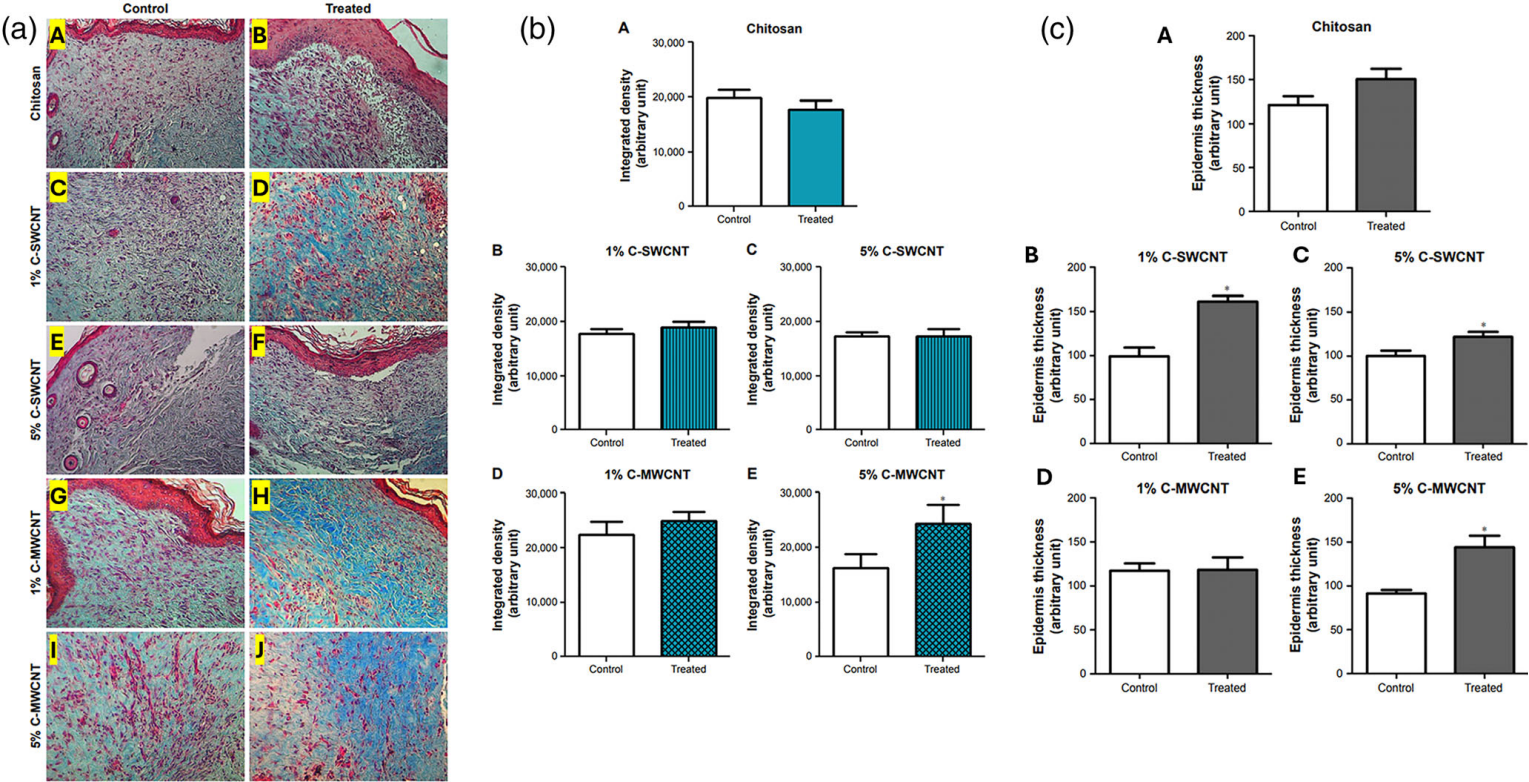
(a) Treatment with 5% chitosan‐multi‐walled carbon nanotubes (C‐MWCNTs) best improved the fibrotic processes in wound healing. At the end of the 9‐day treatment period, Masson trichrome stains were collected for sectioned full‐thickness skin tissue samples at the site of the healing wounds. The bluish‐green color represents the stained collagen. Magnification 100×. (A, C, E, G, I) Internal controls of the corresponding treatment conditions; (B) after treatment with chitosan; (D) after treatment with 1% C‐single‐walled carbon nanotubes (SWCNTs); (F) after treatment with 5% C‐SWCNTs; (H) after treatment with 1% C‐MWCNTs; (J) after treatment with 5% C‐MWCNTs. (b) C‐MWCNT was the most effective treatment for increasing the amount of collagen deposited in the healing wound. Images of tissue samples stained with Masson's trichrome stain were digitally analyzed using ImageJ's “Color Deconvolution” plugin. (A) C treatment; (B) Treatment with 1% C‐SWCNTs; (C) Treatment with 5% C‐SWCNTs; (D) treatment with 1% C‐MWCNTs; (E) Treatment with 5% C‐MWCNTs. Values are presented as means ± SEM, *n* = 5, in triplicate. *p 0.05 compared to control. Treatment with 5% C‐MWCNTs had a significantly more significant effect than the corresponding internal control. There was no significant difference in the other treatment conditions. (c) 1% C‐SWCNTs, 5% C‐SWCNTs, 1% C‐MWCNTs, and 5% C‐MWCNTs improved the formation of the epithelial layer (A–E). Representative random images of tissue samples stained with Masson's trichrome stain were analyzed using ImageJ software using the “straight line” tool to arbitrarily measure epidermal thickness. Values are presented as means ± SEM, *n* = 5, in triplicate. *p 0.05 compared to control.^159^

Kittana et al.^159^ studied the effect of chitosan hydrogel containing MWCNT and SWCNT on wound healing. In in vitro studies, by placing hydrogels in the external matrix of the skin, they observed that fibroblast cells grew along the axis of the tissue, and the ECM was formed in the presence of the hydrogel containing MWCNT and SWCNT, contracting the tissue and maintaining its structure. In in vivo studies, due to the effective contact between the hydrogel and the wound, wound healing occurred. In the sample containing 5% MWCNT and 1% SWCNT, the formation of fibrotic tissue was reported at 80%, which was more than in the other samples, and showed a smaller and drier wound because they regenerate epidermal and fibrotic tissue and increase the deposition of collagen. The higher amount of collagen deposition in the sample containing C‐MWCNT (~120%) indicates an increase in inflammatory cells in the wound. It seems that the increase in inflammatory cells was in line with wound healing activities. In addition to the regeneration of the connective tissue layer, the regeneration of the epithelial tissue layer in the C‐SWCNTs and C‐MWCNTs samples was also seen, with an increase in the thickness of the epithelium (Figure 8a–c). In this study, the wound healing process, which includes the activation of inflammatory cells and fibroblast cells, followed by collagen secretion and ECM synthesis to form fibrous tissue, and then the expansion of the epidermis thickness to create a protective layer, occurred faster in the sample containing 5% MWCNT.

One of the most fundamental problems of CNTs is their hydrophobicity,^154^ which causes their insolubility in water due to long‐range Van der Waals forces and agglomeration. Although they can be dispersed by ultrasonic method, as soon as it stops, the particles settle again. Techniques such as creating supramolecular complexes or forming suspensions in toluene, dimethylformamide (DMF), tetrahydrofuran (THF), chemical modification with pyrrole compounds, fluorination, and the use of surfactants can moderate this problem, in which case, by increasing the contact surface of carbon nanomaterials with bacteria, the antibacterial activity also increases.^103^, ^165^, ^166^

### 3.4 Carbon nanofibers

In 1879, Thomas Edison used CNFs as filaments in light bulbs, which he made by dissolving cellulose, extruding it, and finally carbonizing it. Carbon fibers contain at least 97% by weight of carbon in the form of graphite crystals, which gives them a high anisotropic structure. Carbon fibers with a high Young's modulus of 106–406 GPa and yield strength of 1.5–5.65 GPa are suitable for skin and tendon tissue engineering.^167^ Also, the conductivity of CFs enables the formation of vessel‐like structures for angiogenesis in skin tissue engineering.^168^ Carbon nanofibers are one‐dimensional filaments of the sp^2^ group^167^ with a diameter of 200–70 nm and a length of 200–50 μm,^169^ which are seen in cylindrical or conical shapes.^170^ These carbon nanofibers are synthesized by electrospinning and chemical vapor deposition (CVD).^167^ In the CVD method, the structure of nanofibers is controlled by the type of catalysts, such as iron, nickel, or chromium, which are capable of producing metal carbides.^167^ In the electrospinning method, oxidative stabilization and carbonization thermal treatment are performed, both affecting the final carbon nanofiber morphology; for example, in oxidative stabilization, the melting of fibers is prevented to maintain the amount of carbon.^171^ In carbonization, the fibers are subjected to a neutral gas under a temperature of 1000°C to maintain purity and crystallinity and ultimately reduce the diameter of the fibers.^167^ Antibiotic resistance against *P. aeruginosa*,^172^ which is one of the common bacteria causing skin infections in burns,^173^ cystic fibrosis, cancer, trauma, and sepsis, is one of the challenges of treating skin diseases.^172^

In a study conducted by Ashfaq et al.,^173^ they synthesized a PVA‐cellulose acetate phthalate(CAP) film containing activated carbon microfibers and carbon nanofibers dispersed in Cu/Zn nanoparticles to prepare a wound dressing that prevents the activity of *P. aeruginosa* bacteria. The hemostatic and antibacterial properties were attributed to the presence of PVA‐CAP and copper nanoparticles, respectively. They concluded that the presence of carbon nanofibers and microfibers was a support to control the release of nanoparticles and platelet aggregation, which led to clot formation and initiation of wound healing. Biochemical and cellular tests of the dressing showed appropriate behavior regarding the activation of platelets, division of macrophage cells, and formation of clots, which caused the division of endothelial and epithelial cells and the formation of connective tissue.

**FIGURE 9:**
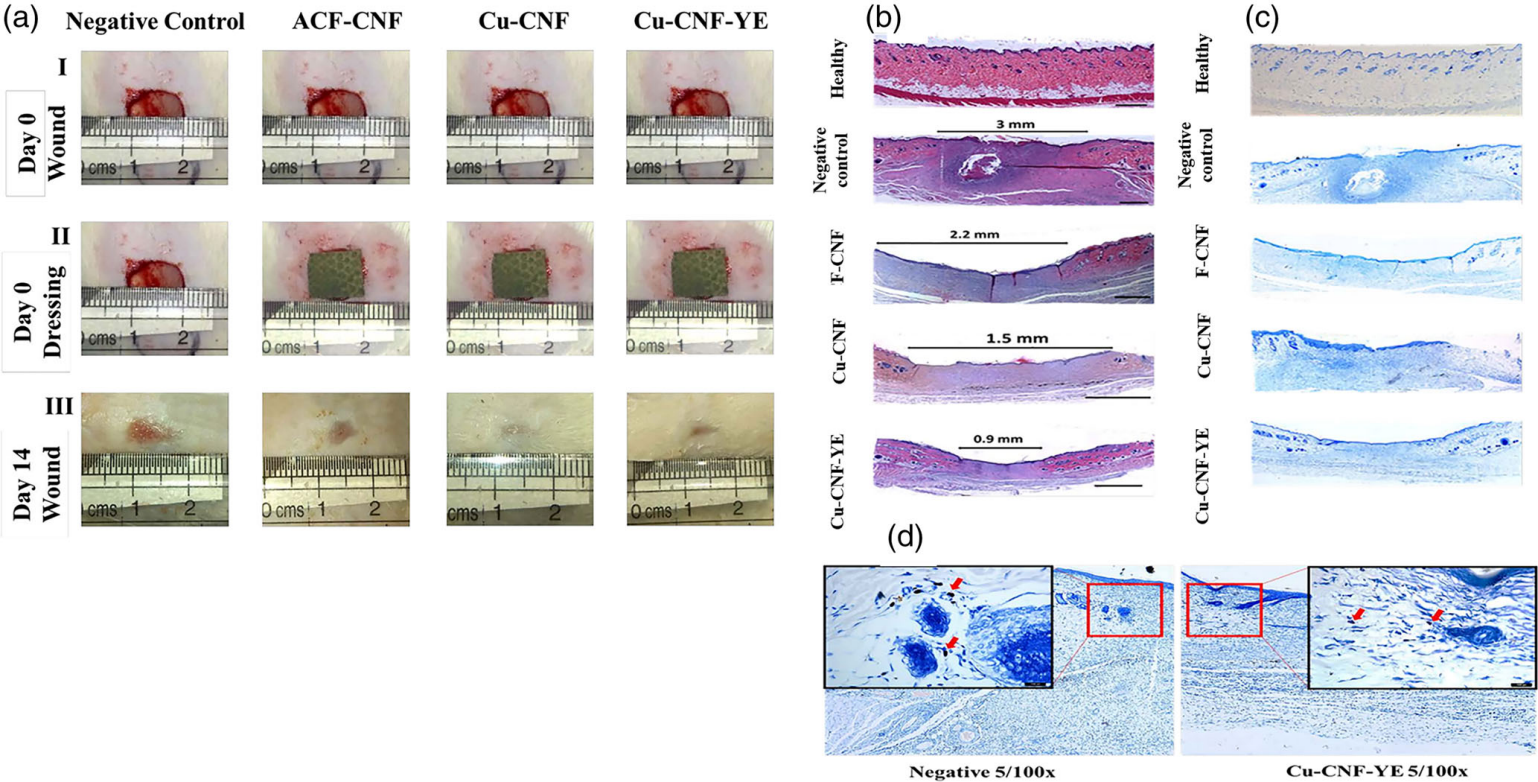
(a) Images of a diabetic wound on Days 0 and 14 showing (I) the resulting defect area, (II) the applied dressing, and (III) gross changes in size/area and degree of wound closure between different groups. (b) Histological cross‐section of healthy and wound skin of a diabetic animal on Day 14 (scale bar = 500 μM). (c) Toulidine blue staining of healthy and diabetic wound skin on Day 14. (d) Toulidine blue stained images compared at 5× magnification along with the inset at 100× magnification showing significant mast cell infiltration at the wound site.^174^ Cu‐CNF‐YE, copper nanoparticles dispersed on carbon nanofibers stabilized with yeast.

Bhadauriya et al.^174^ utilized the same properties of carbon nanofibers and their low toxicity to control the release of copper nanoparticles. To create wound dressings for diabetic wounds that lead to bacterial infection, they made copper nanoparticles dispersed on carbon nanofibers stabilized with yeast (Cu‐CNF‐YE). In histopathological analysis, they observed the formation of neo‐epidermis tissue toward the inside of the wound after 14 days in mouse skin, as well as neovascularization and the formation of multiple hair follicles, and showed a ~95% wound healing rate. While in the control group, the wound was still open, and scar tissue was visible. Additionally, in Cu‐CNF‐YE, only 0.9 mm of scar tissue was formed, and the fibroblastic wound was closed with the best performance compared to the other groups due to the absorption of other cells. Mast cells, which are responsible for causing inflammation in the wound, also decreased, indicating the beginning of tissue repair. Furthermore, the antibacterial properties of yeast and copper nanoparticles in carbon nanofibers effectively prevented the growth of *E. coli* and *S. aureus* strains (Figure 9a–d).

**FIGURE 10:**
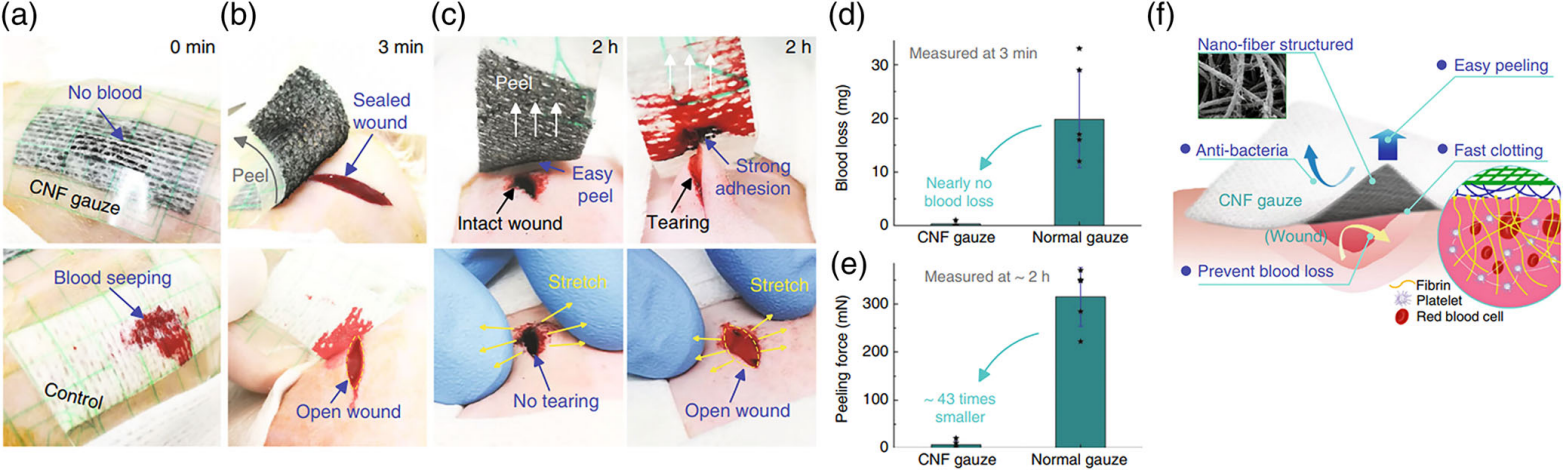
In vivo animal testing. (a) The plaster‐like gauzes were patched onto the incisions on the rat's back. The control cotton gauze quickly became wet, while the carbon nanofibers (CNF) gauze prevented blood loss. (b) Removing the gauze after 3 min to measure blood loss; the CNF gauze helped form a gel‐like clot and seal the wound properly. An open wound was observed under the control gauze. (c) Peeling the gauze after about 2 h to measure the peeling force; the CNF gauze was easily peeled off, and gentle stretching of the wound did not cause wound rupture or bleeding (Movie S1). In contrast, peeling off the ordinary gauze resulted in wound rupture and bleeding (Movie S2). (d) The CNF gauze minimized blood loss (*n* = 6). (e) The peeling force of the CNF gauze was significantly lower than that of the normal gauze (*n* = 5). (f) Schematic representation of CNF hemostatic gauze/patch for wound treatment. The data in (d) and (e) are shown as mean ± SD; the error bar represents SD, and individual data points in (d) and (e) are represented by black stars.^175^

Li et al.^175^ synthesized superhydrophobic hemostatic dressings by spraying polytetrafluoroethylene (PTFE)/CNFs and polydimethylsiloxane (PDMS)/CNFs composites on the Ti6Al4V surface to overcome challenges such as delay in clot formation and blood loss and easy removal of the dressing without bleeding. The contact angle of the CNFs/PTFE‐Ti surface and CNFs/PDMS‐Ti surface was reported as 162 ± 2.9° and 154.9 ± 0.6°, respectively. In the Fibrin ELISA and animal tests, they observed that carbon nanofibers accelerate the growth of fibrins, resulting in faster clot formation and less blood loss (0.3 mg for CNF gauze and 19.8 for normal gauze), and the average maximum peeling force for CNF gauze was about 43 times lower than normal gauze. Because CNF reduces adhesion and makes the clot easier to separate, it prevents tissue damage and rebleeding and allows the wound to heal more quickly. In the results of the antibacterial test, due to the choice of hydrophobic materials for making wound dressings and the roughness of the surface of carbon micro/nanofibers, they observed less adhesion of bacteria (Figure 10a–f).

Due to its homeostatic and hydrophobic properties, it can be a suitable option for producing nano‐wound dressings. However, it should be considered that CNFs can create debris due to the shear stress caused by the weak force between the graphite planes, so ways to improve the mechanical properties should be considered. To illustrate, reducing the fiber diameter can improve tensile strength and flexibility, and it also increases the ratio of surface area to volume and reduces structural defects.^167^

### 3.5 Nanodiamond particles

Diamond nanoparticles contain an organized core^176^ of sp^3^ carbon atoms with covalent bonding (tetrahedral hybridization).^177^ Based on the size of the particles, they are divided into the groups of diamond crystal nanoparticles, diamonds, and ultra‐nanocrystalline diamond particles, which are 10–100, 1–2 nm, and 10–2 nm, respectively.^178^ From a schematic point of view, they are generally round or multifaceted.^176^ Due to their biocompatibility and appropriate surface chemistry, biological conjugation is possible^141^ and, as a result, their use as a drug carrier for skin diseases as an anti‐scar agent.^176^ Due to its high surface‐to‐volume ratio, diamond nanoparticles allow drug loading in large quantities and improve the drug's stability time.^176^ Nanoparticles such as nanodiamonds (NDs) have antibacterial properties by destroying the membrane of bacteria and then penetrating the bacteria, which causes bacteriostatic and bactericidal effects by leaking the cytoplasmic components of the bacteria.^179^, ^180^ Along with their suitable properties for drug delivery and tissue engineering, toxicological studies regarding their widespread use should be conducted due to the possibility of their passing through the cell membrane.^178^ To date, methods such as dynamic synthesis using explosion techniques, high pressure and high temperature (HPHT), ball milling of diamond microcrystals, laser erosion, and chemical vapor deposition have been used to synthesize diamond nanoparticles in different sizes.^181^ In the meantime, the dynamic synthesis method using explosion techniques produces ultra‐nanocrystalline diamond nanoparticles with a size of 4–5 nm, which are used for commercial biomedical applications.^178^ Due to its low cost, simple equipment, fast reaction speed, and high biocompatibility, this method has drawn much attention for synthesizing explosive diamond nanoparticles and its use in nanomedicine science.^181^ When diamond nanoparticles are exposed to electron beam radiation due to their photoluminescence properties,^178^ vacancy defects called nitrogen‐negative central vacancies are created in them.^182^

**FIGURE 11:**
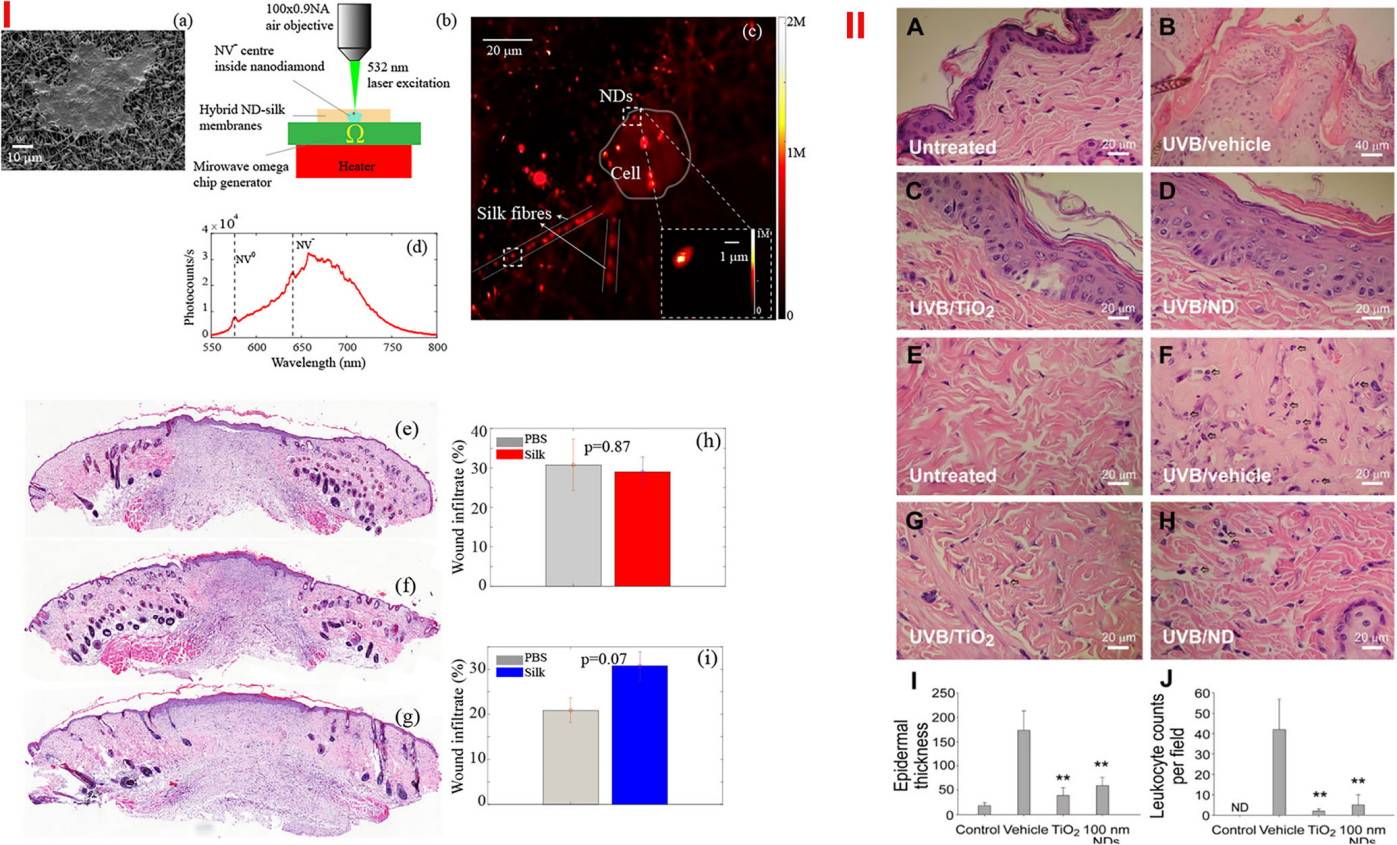
(I) (a) Scanning electron microscope images of human epidermal keratinocyte cells (HaCaT) cells grown on nanodiamond particles (ND) silk fiber membranes after 48 h of cell culture. (b) Schematic representation of the setup for optical excitation of negatively charged nitrogen‐vacancy (NV−) centers and temperature measurement in silk‐embedded NDs. A MW field is applied using an Omega chip resonator, and the temperature is varied using a thermoelectric Peltier heater. (c) 100 × 100 μm^2^ fluorescence map of the cell‐cultured ND‐silk membranes on a glass coverslip. The central rounded area is one of the solid cells growing on the hybrid membrane. The solid straight lines represent the silk fibers with embedded NDs. The dashed boxes show two representative NDs, and the inset in the solid box shows a magnified 10 × 10 μm^2^ scan of the selected area of interest. (d) Fluorescence spectrum obtained from fluorescent NDs confirming the presence of NV centers. Representative histological cross‐sections of the hematoxylin and eosin‐stained wounds for (e) PBS‐, (f) silk membrane‐, and (g) ND‐silk membrane‐treated wounds at Day 10 post‐wounding. Wound cell infiltrates calculated for each wound as a percentage of the total wound granulation area: (h) PBS versus silk membranes and (i) PBS versus ND‐silk membranes. *p* Values were derived from an unpaired *t*‐test for (h, i). All data are presented as mean ± SEM (standard error of the mean) with *n* = 3–4 mice.^183^ (II) NDs alleviate UVB‐induced skin hyperplasia and leukocyte infiltration. Histological examinations of the epidermis (a–d) and dermis (e–h) showed the changes before (a, e) and 3 days after UVB irradiation (UVI 6, 20 min per day, three cycles; [135.9 mJ/cm^2^ /day × 3 days]), with and without protection using 2 mg/cm^2^ TiO_2_ and 100 nm ND nanomaterials (b–d, f–h; H&E staining, ×400, scale bars in (a) and (c–h) = 20 μm and in b = 40 μm). Epidermal thickness was quantified as indicated (i). The infiltrated leukocytes (indicated by arrows) were found in the dermis, particularly in the vehicle group (f–h); Quantified results are shown in (j). *n* = 9, **p < 0.01, a significant improvement over vehicle groups. Data are means ± SD.^186^

To heal the wound and measure its temperature, Khalid et al.^183^ used the electrospinning method to create a silk‐ND membrane containing nitrogen‐central vacancy. The presence of negatively charged nitrogen‐vacancy (NV−) in NDs showed optical magneto‐resonance (ODMR). It acts as a nanoscale thermometer to measure wound temperature, an essential factor in inflammation or skin infection. They observed that nitrogen‐central vacancies with a negative charge (NV−) in NDs caused a more significant change in frequency and displayed accurate temperature, creating sensitivity for wound temperature measurement. This makes the wound healing traceable, allowing for determining the right time to change the dressing. For in vitro studies, human keratinocyte cells were cultured on the membranes, and after 48 h, the adhesion and growth of keratinocyte cells were observed and reported as 85%–90%. After 48 h, the cells were fixed and examined through a custom‐made confocal fluorescence scanning microscope, resulting in a 100 × 100 μm square fluorescence map of the cell‐cultured ND‐silk. In the fluorescence map, they observed a keratinocyte cell on the ND‐silk membrane (before fixation). They also confirmed the presence of NV centers by preparing the fluorescence spectrum. To investigate wound closure in an animal model after 10 days, they found that the presence of NDs in the silk‐ND membrane did not have a significant difference in wound closure compared to the PBS control group, which could be due to the faster degradation of silk and the decrease in the membrane's ability to close the wound. Biological diversity and the possibility of causing a partial inflammatory response of NDs were also observed; however, they do not cause severe inflammation and do not disrupt the natural wound healing process. In the antibacterial test, 99% and 97% of dead *P. aeruginosa* and *E. coli* bacteria were observed on the surface of the silk membrane and silk‐ND membrane, respectively (Figure 11I).

Houshyar et al.^184^ Polycaprolactone‐ND nanofibers were produced by electrospinning to investigate the effect of different percentages of NDs on mechanical, thermal, and morphological properties. With the increase of NDs, the fiber diameter increased. In addition, due to the high thermal conductivity of NDs in the thermogravimetric analyzer (TGA) test, thermal degradation decreased with the increase in NDs. ND had caused thermal stability of the nanocomposite because ND causes faster heat loss, similar to the findings obtained in the study of Wu et al.^185^ According to the hydrophobicity of PCL and the hydrophilicity of NDs, with the addition of NDs, they saw an increase in the hydrophilicity of nanofibers and the adhesion and growth of mouse epithelial cells even in samples with a higher concentration of NDs, which indicates that they do not cause harmful effects on cells. In addition, with the rise in the concentration of NDs, there is a decrease in the tendency of *S. aureus* bacteria to attach to the surface and also an increase in the hydrophobicity of the surface, which causes an antibacterial effect.

To synthesize a bacterial CS/cellulose wound dressing, Mahdavi et al.^187^ found that the mechanical strength increases with the addition of NDs, and the hydrophilicity of the nanofibers is controlled and decreased. Additionally, with an increase in the percentage of NDs, due to the formation of ND clusters and agglomeration, the viscosity decreases, and the diameter of the fibers decreases. On the other hand, by increasing the percentage of NDs beyond 2 wt%, the growth of fibroblast cells decreased due to agglomeration; however, the viability rate reported 75%–90% for samples with different concentrations of NDs (1–3 wt%).

Titanium nano oxide and zinc nano oxides are used in skin products as protective and cosmetic agents due to their photocatalytic nature, which is not beneficial for the skin. However, this challenge only exists for NDs. Wu et al. investigated this and observed that NDs protect keratinocyte cells, fibroblasts, and the skin of C57BL/6J mice against UVB, which are UV waves, compared to titanium nano oxide and zinc nano oxide. Also, in a comparison between 100‐nm NDs and 5‐nm NDs, it was observed that 100‐nm NDs do not penetrate the deep layers of the skin. These NDs can also convert UV waves into harmless infrared molecules, thus providing greater protection against UV light than the skin. In the in vivo test, by measuring the hyperplasia factors of granulosome, spinosome, and leukocyte infiltration, which occur in the skin when exposed to UV radiation, they observed that in the mouse model treated with ND, these cases were milder, and the number of infiltrating leukocytes in the dermis was lower than the control sample (Figure 11II).^186^

Finally, along with positive features such as excellent biocompatibility, absorption, and high hydrophilicity, which are suitable for drug release applications and skin care products, it should be considered that because the toxicity of NDs depends on their concentration, there is a risk of causing cytotoxicity, genotoxicity, and oxidative stress, which should be paid attention to.^188^ However, according to Moradi et al.'s study,^189^ NDs can send a cell death signal to skin cancer cells without causing high cytotoxicity in other tissues.

### 3.6 Fullerene

Like diamond nanoparticles, fluorenes are zero‐dimensional carbon nanomaterials containing 60 sp^2^ carbon atoms that have created a spherical shell structure.^190^ A significant challenge of fluorenes is their insolubility in polar solvents, which, due to their surface chemistry, are prone to forming bonds^191^ that can be functionalized with groups such as NH_2_, ‐OH, and ‐COOH.^192^ This action has increased their solubility,^191^ which makes fluorenes suitable candidates for drug transport, along with factors such as high pore area and volume.^192^ Fluorenes produce ROS due to white light irradiation in aerobic conditions, inhibiting their activity in reaction with the cellular components of microbes.^191^ Fluorenes are also used in the medical field as antibacterial, antioxidant, human immunodeficiency virus (HIV) inhibitors, and light‐sensitive agents in photodynamic therapy.^193^

In addition to the challenge of the insolubility of fluorene in water, which can be overcome by methods such as Bingel‐Hirsch, polyhydroxylated, and Prato reactions, it should be noted that due to their small van der Waals diameter (1.1 nm), they can penetrate the cell membrane. Furthermore, they have an accumulation that affects cellular behavior.^193^

Vlasov et al.^194^ investigated the therapeutic effect of fluorine C60 colloidal solution on cold sores, mainly caused by cryosurgery. After introducing the colloidal solution of fluorine C60 into the abdominal cavity of mice samples for 5 days from the onset of frostbite, they observed that after 21 days of treatment, there was an increase in the proliferation of epithelial cells and an increase in the thickness of the epithelial layer. Also, over time, the thickness of necrotic tissue was reduced, and the thickness of the granulation layer increased. They concluded that hemodynamic disorders and cell infiltration were less common in mice, and as a result, the thickness of secondary necrotic tissue was also reduced. These events happened because of the antioxidant activity of fullerenes, which prevent the oxidation, inflammation, and secondary damage and enhance the activity of fibroblasts and deposition of collagen that is essential for skin repair after cryodestruction.

**FIGURE 12:**
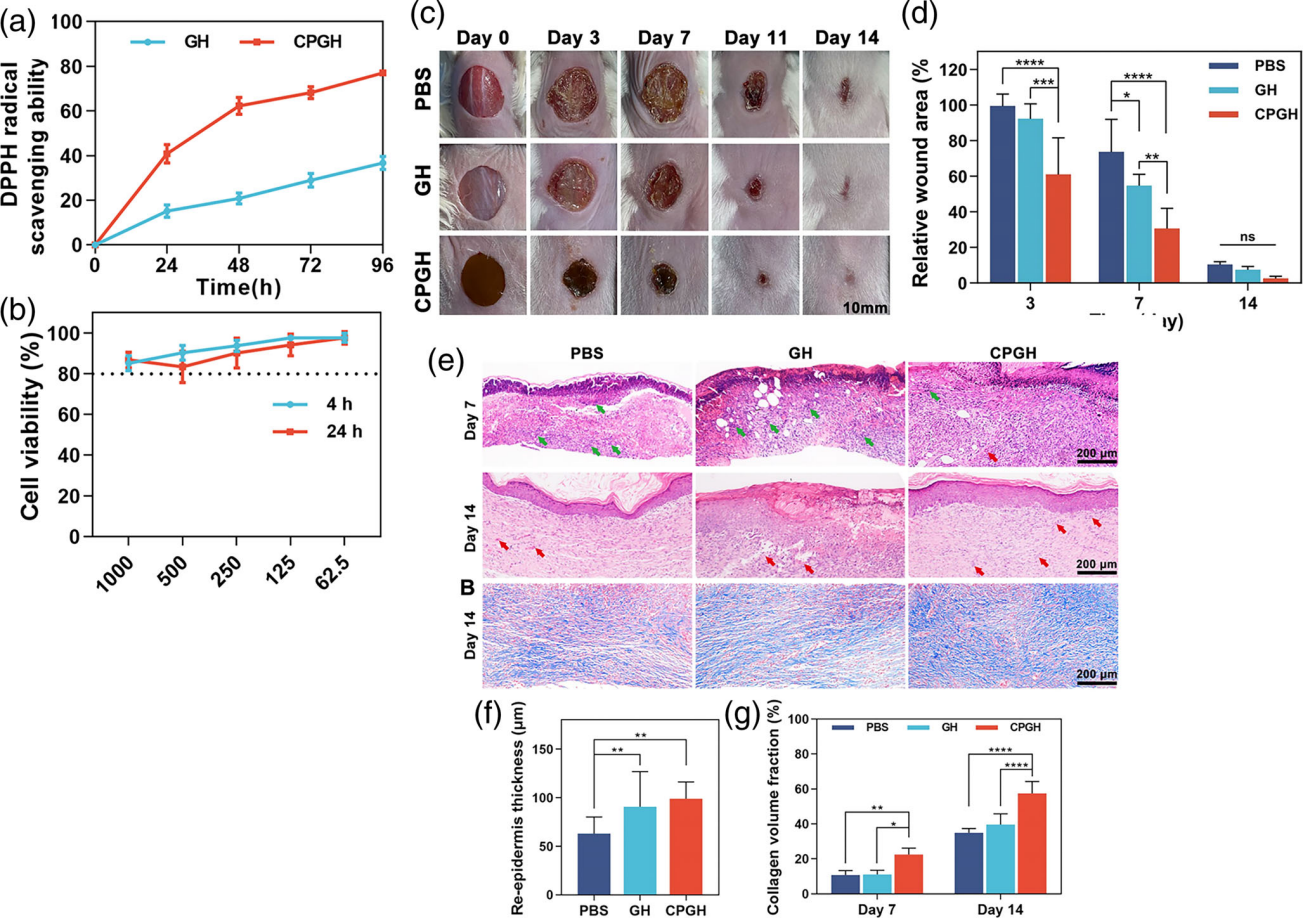
Antioxidant capacity and in vitro biocompatibility testing of hydrogels. (a) 2,2‐diphenyl‐1‐picrylhydrazyl free radical (DPPH) radical scavenging percentage of GelMA, C60‐PDA/GelMA. (b) Cell viability of L929 cells after 4 and 24 h incubation with C60‐PDA of different concentrations. Wound closure analysis in vivo. (c, d) Photographs of wounds on Days 0, 3, 7, 11, and 14 after treatment. (d) Wound closure analysis from the existing wound area on Days 3, 7 and 14 after treatment. (*n* = 3, *p < 0.05, **p < 0.01, ***p < 0.001 and ****p < 0.0001). Histological analysis of the wound healing effect. (e) H&E staining of skin wound sections at 10× magnification on Days 7 and 14 after treatment. Red arrow: blood vessels; green arrow: inflammatory cells. (f) Masson's trichrome staining of wound tissue on Day 14 after treatment. (g) Quantification of re‐epidermal thickness at Day 14 post‐treatment. (d) Quantitative analysis of collagen content (%) after 7 and 14 days of treatment. *p < 0.05, **p < 0.01, ***p < 0.001 and ****p < 0.0001.^195^

To reduce the oxidative stress in the wound, Chen et al.^195^ made an antioxidant GelMA‐fluorene‐PDA (C60‐PDA/GelMA) nanocomposite wound dressing. They observed that fewer DPPH‐free radicals (nearly 0%) were produced in samples containing C60‐PDA and had increased antioxidant activity by scavenging ROS such as hydroxyl and superoxide. In cell studies, increasing amounts of C60‐PDA increased the percentage of L929 cells and achieved biocompatibility and antibacterial activity, also induced a protective effect, and reduced oxidative stress inside cells. In animal studies, they found that the wound closed faster in the C60‐PDA/GelMA hydrogel than in the sample without C60‐PDA, which was accompanied by neovascularization, the appearance of blood vessels, reduction of inflammatory cells, and formation of granulation tissue, collagen production (57.5%), and an increase in the thickness of the epidermis (Figure 12a–g).

### 3.7 Carbon nano‐onions

Carbon nano‐onions (CNOs) are small zero‐dimensional spherical or polyhedral compounds with an onion‐like structure in which multilayer shells surround each other. This onion‐shaped structure consists of at least four concentric shells whose structure is disordered or defected by sp^3^‐hybridized carbon. Their size is between 3 and 100 nm, depending on the synthesis method, and this small size gives them unique physicochemical characteristics. The surface of these spherical structures is around 300–600 m^2^/g, which is higher than that of other carbon allotropes such as graphite, carbon nanotubes, or graphene. Compared to other sheet‐like carbon nanomaterials (such as graphene), CNOs have a more stable mechanical structure due to their spherical nanostructures. Due to their biocompatibility and chemical stability, they are used in nanomedicine, and to solve problems such as low solubility and dispersion, they can be covalently or non‐covalently modified.^196^

In Castro et al.'s study,^197^ by implanting CS‐PVA‐oxidized CNO nanofibers in the subdermal area of Wistar rats, they observed that increasing the amount of oxidized CNO increases the crystallinity and biocompatibility without affecting the implantation process. During the first 30 days, fibrous scar tissue was mostly around the implantation site. However, no severe inflammatory response or tissue necrosis was observed in the next 60 days, indicating that CNO, due to its oxygen‐containing functional groups, interacted well with the wound environment and stabilized the structure without causing an aggressive reaction. Also, the high crystallinity of CNO increased its stability against enzymatic degradation and made it more suitable for long‐term use in tissue engineering. Implantation of CNO‐containing nanofibers under physiological conditions resulted in the growth of new blood vessels, fibroblast activity, and the growth of healthy adipose tissue, indicating the biocompatibility and stability of the scaffold.

**FIGURE 13:**
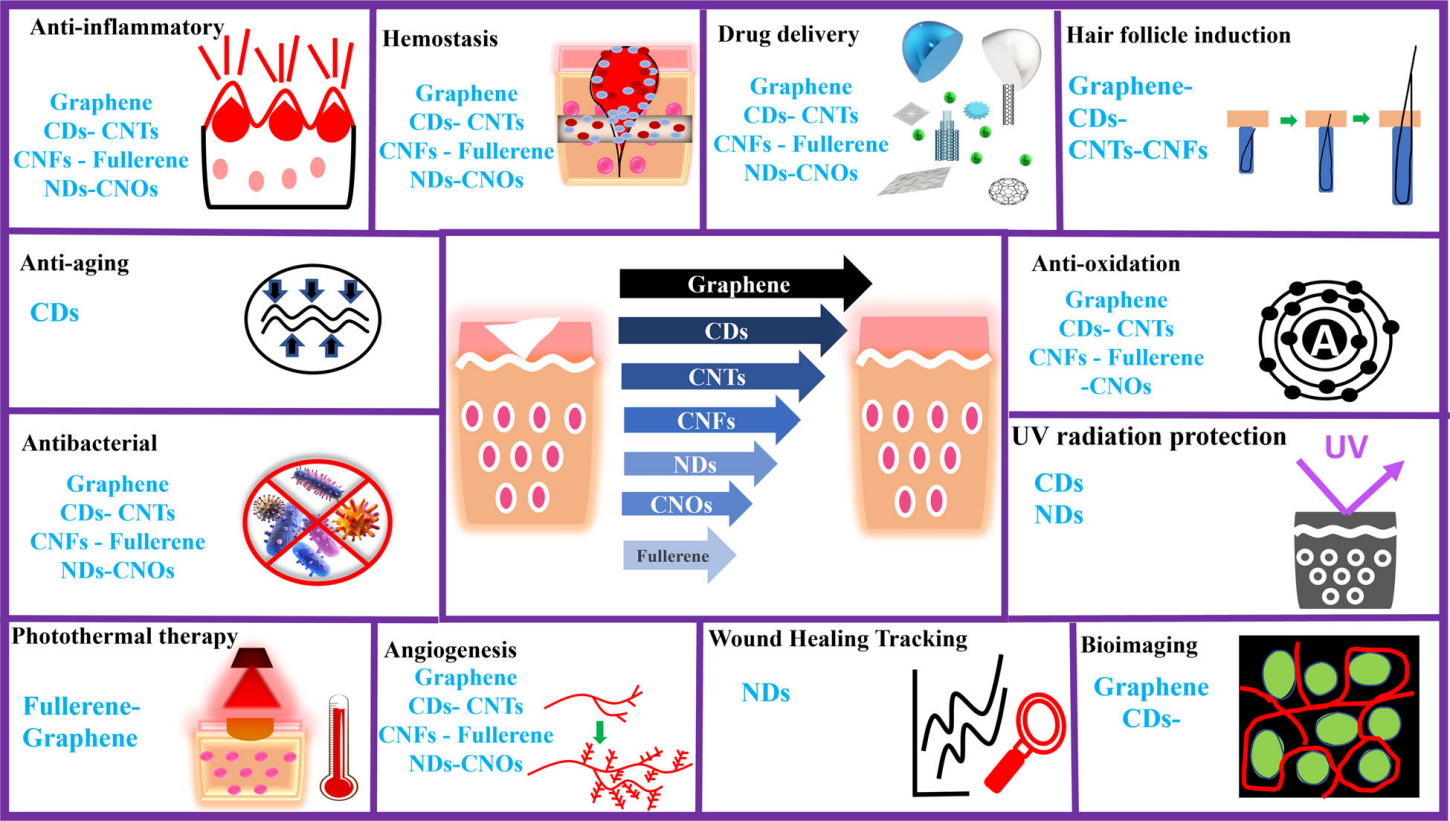
Carbon nanomaterials in wound healing and medicine. CDs, carbon dots; CNFs, carbon nanofibers; CNTs, carbon nanotubes; CNOs, carbon nano‐onions; NDs, nanodiamond particles.

Figure 13 below provides a summary of the functions of carbon nanomaterials investigated in detail in this study, specifically in the context of wound healing and skin tissue engineering.

In the following, we will discuss other studies about using carbon nanomaterials in wound healing (Table 4).

**TABLE 4:** Some studies on the use of carbon nanoparticles in wound healing.

Carbon‐based nanomaterials	Description	Results	References
NDs	Chitosan/acterial cellulose composite films containing nanodiamonds	NDs increased young modulus Reduction of crystallinity in scaffold and thus a lower tensile strength. Reasonable cytocompatibility of fibroblast L929 cells	^198^
	Doxorubicin‐loaded nanodiamonds/cellulose nanocomposite membranes	No toxicity observed for Hela cell More release of doxorubicin at lower pH	^199^
CNFs	Copper/zinc bimetal nanoparticles‐dispersed carbon nanofibers	Suppression of *Escherichia coli*, *Staphylococcus aureus*, and MRSA bacteria growth Prolonged release of the CuNPs No toxicity, with 0.35% hemolysis	^200^
	Calcium Alginate‐carbon nanofibers composites films	Antibacterial activity of CNFs Antibacterial effect for multidrug resistant *Staphylococcus epidermidis* bacteria Non‐toxic nature of CNFs for human keratinocyte HaCaT cells	^201^
CNTs	*N*‐carboxyethyl chitosan (CEC)‐benzaldehyde‐terminated pluronic F127/carbon nanotubes (PF127/CNT)	Increasing CNT content increased mechanical properties and water absorbency CNT‐induced suitable photothermal antimicrobial activity CNT‐induced good conductivity CNT‐induced stable hemostatic properties Good biodegradability Wound closure healing by collagen deposition and angiogenesis	^202^
	Bacterial cellulose‐MWCNT composite films	Antibacterial activity MWCNT caused faster healing of the diabetic wound by way of decreasing the expression of IL‐1α and TNF‐α and increasing the expression of VEGF	^203^
	Gelatin‐grafted‐dopamine (GT‐DA) and polydopamine‐coated carbon nanotubes (CNT‐PDA) composite hydrogel	Antimicrobial activity by adding antibiotic doxycycline Physical, mechanical, and swelling properties of the scaffold changed by the concentration of CNT‐PDA Good in vitro biocompatibility for L929 cells after 5 days Wound closure, collagen deposition after 14 days	^204^
	GelMA/CNT conductive macroporous nanocomposite hydrogels	Increasing mechanical strength, shape recovery, and electrical conductivity by adding CNTs Good NE‐C4 compatibility Antimicrobial activity Wound healing in 2 weeks in murine	^205^
CDs	Citric acid‐based N‐doped CNDs AND e N.S‐doped CNDs	N‐doped CNDs and N.S‐doped CNDs caused antioxidant and anti‐inflammatory abilities in scaffold Low cytotoxicity and blood compatibility Wound closure in the N‐doped CNDs group from day two and complete healing in 8 days	^206^
	Cerium‐doped carbon dots nanoplatforms	Good hydrophilicity, biocompatibility, and excellent photostability under UV excitation Special antibacterial activity under UV excitation Fibroplasia and angiogenesis effects Ce‐CNDs treated wounds showed fewer inflammatory	^207^
	Black phosphorus nanosheets loaded with cationic carbon dots.	Cytocompatibility for L‐929 cells and hemocompatibility Antibacterial abilities Observing dermal fibroblasts and new blood vessels by laser irradiation	^208^
	Spermidine‐CDs‐acrylic acid‐pectin (SCDs‐AP) hydrogel	High antibacterial activity owing to the positively charged CDs Good tissue adhesiveness and fibroblast cell adhesion	^209^
CQDs	Chitosan‐carbon quantum dot‐titanium dioxide‐graphene oxide (CS‐CQD TiO_2_‐GO) nanofibrous mats	Antibacterial activity of nanofibers Cell viability of mouse fibroblast NIH3T3 cells The highest rate of wound healing, good collagen organization, less inflammatory responses, and better reepithelization in CS‐CQD‐TiO_2_‐GO nanofibrous mats after 14 days	^210^
	Quaternized carbon quantum dots(Q‐CQDs) derived from curcumin and 2,3‐epoxy propyl tri methyl ammonium chloride (GTA)	Antibacterial activity of Q‐CQDs by ROS production Low hemolysis rate Biocompatible for 293T, Hep G2, B16F1, and Bewo cells Low inflammation Insignificant toxic results on sensitive organs	^211^
rGO	polyvinyl alcohol/polyvinylpyrrolidone/nano‐rGO hydrogel	Increasing rGO caused decreasing the water absorption rate The addition of nano‐rGO increased the stiffness and modulus Adding nano‐rGO increased their antibacterial characteristics. No toxicity on the L929 fibroblast cell	^212^
	pH‐sensitive rGO/arabinoxylan/chitosan composite	Sharp edges of the rGO and hydrophobicity of the matrix resulted in better antibacterial activity Increasing the amount of rGO improved cell adhesion and proliferation MC3T3‐E1 cells had appropriate cell viability and cylindric morphology on the scaffold	^213^
	Polyvinylpyrrolidone‐eggshell membrane‐reduced graphene oxide nanofibers	Increasing rGO content increased degradation, mechanical strength and resistance to deformation, and reduced swelling ratio Increasing rGO content in the sample increased cytotoxicity due to the agglomeration toxicity effect	^214^
	Sodium carboxymethyl cellulose—(rGO) hydrogels	rGO hydrogel inhibited the formation of *S. aureus* and *P. aeruginosa* biofilm. rGO hydrogel, even at high concentrations, showed low cytotoxicity for fibroblast cells after 24 h	^215^
	Polydopamine‐reduced GO (pGO)‐incorporated CS and silk fibroin (SF) (pGO‐CS/SF) scaffold	Scaffolds reinforced with pGO had excellent mechanical strength and stability in water pGO improved electrical conductivity and dispersion pGO caused intracellular antioxidant activity on macrophages pGO reduced the size of granulation tissue inflammatory cells and caused a regular arrangement of collagen	^216^
GO	Chitosan/gelatin/graphene oxide nanofiber	Increasing the porosity up to 90% by incorporation of GNS within the nanofibers GNS reinforcement increased the antibacterial Good cell migrations and rapid wound‐healing activity	^217^
	Bacterial cellulose/gelatin crosslinked/graphehe oxide hydrogels	Antibacterial activity Less than 0.5% hemolysis rate Enhanced proliferation and cell viability of 3T3 cells with improving cell adherence Increasing GO amount caused: Decreasing hydrophilicity Increasing swelling and biodegradation rate	^218^
	Carrageenan/polyvinyl alcohol/graphene oxide nanofiber	Antibacterial properties Treatment of the inducing scratched wound Histopathological findings showed complete healing after 21 days	^219^
	Chitosan/hyaluronic acid/graphene oxide/copper dressings	Antimicrobial effects by the combination of GO and CuNPs No apparent cytotoxicity for mouse fibroblast cells Re‐epithelialization and organized granulation tissues after 14 days No pathological changes and signs of infection No systemic sepsis in the heart, lung, liver, and kidney Angiogenesis effect of scaffold	^220^
Fullerene	Fullerene nanocomposites encompassed by sodium hyaluronate	Administration of C60 to the edematous and congested ear caused: Reduction of the level of IL‐6 Reduction of the rat ear swelling rate Making an excellent anti‐inflammatory effect	^221^
	Aqueous fullerene C60 dispersion (AFD)	In vivo study showed: No damage to red blood cells No pathological damage in the internal organs Inhibition of TNF‐α, IL‐6, and IL‐1α expression and thus anti‐inflammatory ability of fullerene aqueous Stimulation of hmgb1 and VEGF‐a expression and not formation of scar tissue Interaction of fullerene caused activation of the Nrf2/HO‐1 signaling pathway and, as a result, growth of the antioxidant property	^222^
CNOs	Nanocomposite films of chitosan‐grafted carbon nano‐onions	In vivo studies showed after 30 days of subdermal implantation: Reduced material and increased biosorption for CS: PVA:CS‐grafted‐CNO without immune response. The film enhanced biocompatibility and reabsorption, promoting tissue regeneration.	^223^

Abbreviation: CDs, carbon dots; CNDs, carbon nanodot; CNFs, carbon nanofibers; CNOs, carbon nano‐onions; CNTs, carbon nanotubes; CQDs, carbon quantum dots; GO, graphene oxide; HaCaT, human epidermal keratinocyte cells; MC3T3‐E1 cells, osteoblastic cell line; MWCNT, multi‐walled carbon nanotubes; NDs, nanodiamond particles; NIH3T3, embryonic mouse fibroblast cell line; PVA, polyvinyl alcohol; rGO, reduced graphene oxide; ROS, reactive oxygen species; VEGF, vascular endothelial growth factor.

## 4 CHALLENGES, SOLUTIONS, AND PROSPECTS

Carbon‐based nanomaterials have garnered increasing attention due to their unique physiochemical, mechanical, and biological properties, as reviewed in Section 3. Despite these promising characteristics, several critical challenges hinder their clinical translation and widespread application in wound healing. This section is divided into two subsections: the first highlights key challenges and proposed strategies to address them; the second provides a comprehensive translational outlook incorporating clinical, technological, and regulatory aspects.

### 4.1 Current challenges and proposed solutions

One of the most persistent and widely reported challenges of carbon‐based nanomaterials is their toxicity, which is strongly influenced by factors such as concentration, particle size, shape, surface charge, and degree of functionalization.^224^ These nanomaterials can accumulate in tissues, inducing inflammatory responses, promoting oxidative stress, or even facilitating tumor development.^225^ Most toxicity studies are limited to short‐term evaluations, whereas long‐term in vivo investigations on biodistribution, accumulation, and potential genotoxic effects remain underrepresented.^226^ For instance, CDs exhibit blue‐green fluorescence upon UV irradiation, which, although useful for imaging, may contribute to cytotoxicity via spontaneous fluorescence emission from targeted tissues.^227^

Fabrication challenges are evident in NDs, where achieving diameters below 10 nm remains difficult, yet crucial, due to the enhanced fluorescence, dispersion, and surface reactivity of particles at this scale.^228^ Furthermore, there is a paucity of in vivo biodegradation data for graphene‐based materials and CNTs, despite their frequent classification as non‐toxic.^229^, ^230^ Toxicological concerns for CNTs include excessive ROS production, mitochondrial dysfunction, DNA fragmentation, and potential carcinogenic and teratogenic effects.^230^, ^231^ Similarly, GOs, while promising for wound healing applications, require deeper investigation to elucidate their molecular mechanisms of action and potential for acute and chronic toxicity.^232^

In terms of translational relevance, a major obstacle lies in the species‐specific differences between commonly used animal models (e.g., rodents) and human skin physiology. Rodents exhibit distinct wound healing kinetics and immune responses compared to humans, compromising predictive value.^225^ Large animal models, such as pigs and sheep, have been suggested to bridge this gap, yet remain underutilized in experimental designs.^233^

From a production standpoint, carbon nanomaterials face considerable hurdles related to high manufacturing costs, time‐consuming synthesis protocols, structural inconsistencies, impurities, and toxic waste generation.^9^, ^233^, ^234^, ^235^ These issues are further exacerbated by the absence of globally accepted standardized safety testing frameworks, which contribute to high toxicity assessment costs and limit clinical readiness.^236^

To address these issues, several mitigation strategies have been suggested. Modifying physicochemical properties through surface functionalization can reduce toxicity and improve biocompatibility.^229^, ^237^ The use of polymer stabilizers, metallic coatings, or surfactants can further enhance nanomaterial stability and minimize adverse skin responses.^226^ Additionally, optimizing particle geometry, surface charge, and composition enables better cellular uptake and minimizes ROS production.^230^ Risk reduction also necessitates the development of in vitro–in vivo correlation models, dose–response studies, and long‐term toxicological evaluations. On a broader scale, international regulatory organizations must establish harmonized guidelines for assessing nanomaterial safety in regenerative medicine.^236^

### 4.2 Future directions and translational outlook

The future of carbon‐based nanomaterials in wound healing rests upon a translational paradigm that integrates preclinical innovation with clinical needs, technological capabilities, and regulatory preparedness.

From a clinical perspective, converting promising laboratory results into patient‐centric therapies will require more extensive use of ex vivo human skin models, patient‐derived cells, and biomimetic platforms that more closely mimic human physiology. In addition to improving relevance, these systems can guide the optimization of dosage, administration routes, and therapeutic windows tailored to different types of wounds (e.g., diabetic ulcers vs. surgical incisions).

The advent of smart wound dressings represents a significant leap toward personalized wound management. As discussed earlier, innovative constructs such as CNTs/graphene/GelMA‐based electronic skin (e‐skin),^238^ exhibit sensitivity to strain and humidity, closely mimicking the mechanical behavior of human skin. Similarly, pH‐responsive nanocomposites incorporating GO and phenylboronic acid derivatives for controlled drug release in diabetic wounds,^239^ and the hybrid PA6/PVA/CMWCNT/Cur system responsive to acidic pH,^240^ reflect the growing potential of stimuli‐responsive materials.

**FIGURE 14:**
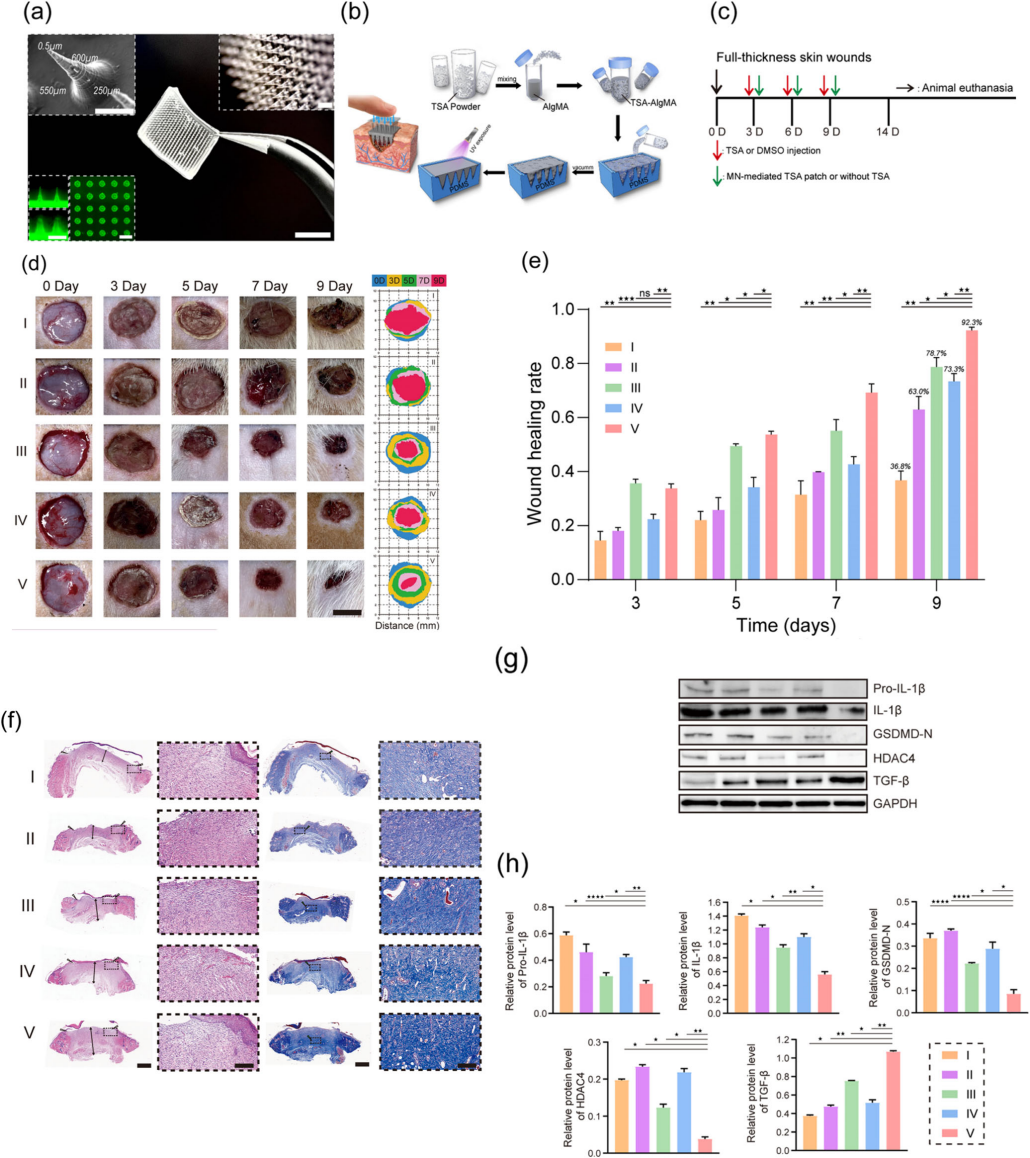
Analysis of the microneedle (MN)‐mediated trichostatin A (TSA) patch on wound healing of diabetic rats. (a) Optical, fluorescence, and SEM images of the microneedle. The SEM image of the individual needle is shown at the top left, and each individual needle has a conical shape with a tip diameter of 5 μm, a height of 600 μm, a base diameter of 250 μm, and a pin spacing of 550 μm; scale bar is 200 μm; Below left are the fluorescence images of the microneedle before or after soaking with PBS for 2 h at 36°C, the scale bars are 200 μm and the five rows of the microneedle array, the scale bar is 550 μm; top right shows the optical image of the part of the microneedle (100×), scale bar is 0.5 mm; The middle Figure shows the optical image of the entire microneedle, the scale bar is 1 cm. (b) Workflow for fabricating the microneedle arrays. (c) Schedule for in vivo evaluation of various therapies. (d) Photos and a graphic illustrating the 9‐day healing process of the wound after various treatments. It turns out that the MN‐mediated TSA patch has the best therapeutic effect. The scale bar is 0.5 cm (*N* = 3). (I) Quantitative analysis of the relative diabetic wound area at different time points (*N* = 3). (f) H&E staining and Masson staining of wound beds on Day 9. Scale bars are 1 mm in 1× and 200 μm in 100×. The results of H&E staining and Masson staining showed that TSA can significantly improve granulation tissue thickness and collagen deposition, especially in the MN‐mediated TSA patch group. WB (g) and relative protein analyzes (h) of Pro‐IL‐1β, interleukin‐1 beta (IL‐1β), Gasdermin D‐N (GSDMD‐N) and histone deacetylase 4 (HDAC4) of diabetic wounds at Day 9, showing that the inflammatory factors and protein levels of the diabetic wound hub gene are lower are in the TSA‐treated groups, particularly in the MN‐mediated TSA patch‐treated group. The protein level of the pro‐healing factor TGF‐β1 is highest in the group treated with MN‐mediated TSA patch, followed by the TSA injection group. (I, II, III, IV, and V denote DM control, DMSO injection, TSA injection, MN‐mediated patch without TSA, and MN‐mediated TSA patch, respectively). Data are presented as mean ± SD (*N* = 3, each experiment is performed three times). *p < 0.05, **p < 0.01, ***p < 0.001, ****p < 0.0001.^242^

The integration of artificial intelligence (AI) is transforming wound care strategies. Predictive models for wound healing progression, such as those applied to diabetic foot ulcers,^241^ rely on machine learning algorithms that can classify and forecast healing outcomes with high precision. The identification of hub genes for diabetic wound therapy using bioinformatics and AI‐guided MN‐mediated TSA patch design^242^ has been graphically summarized in Figure 14a–h, showing enhanced healing metrics and reduced inflammation.

**FIGURE 15:**
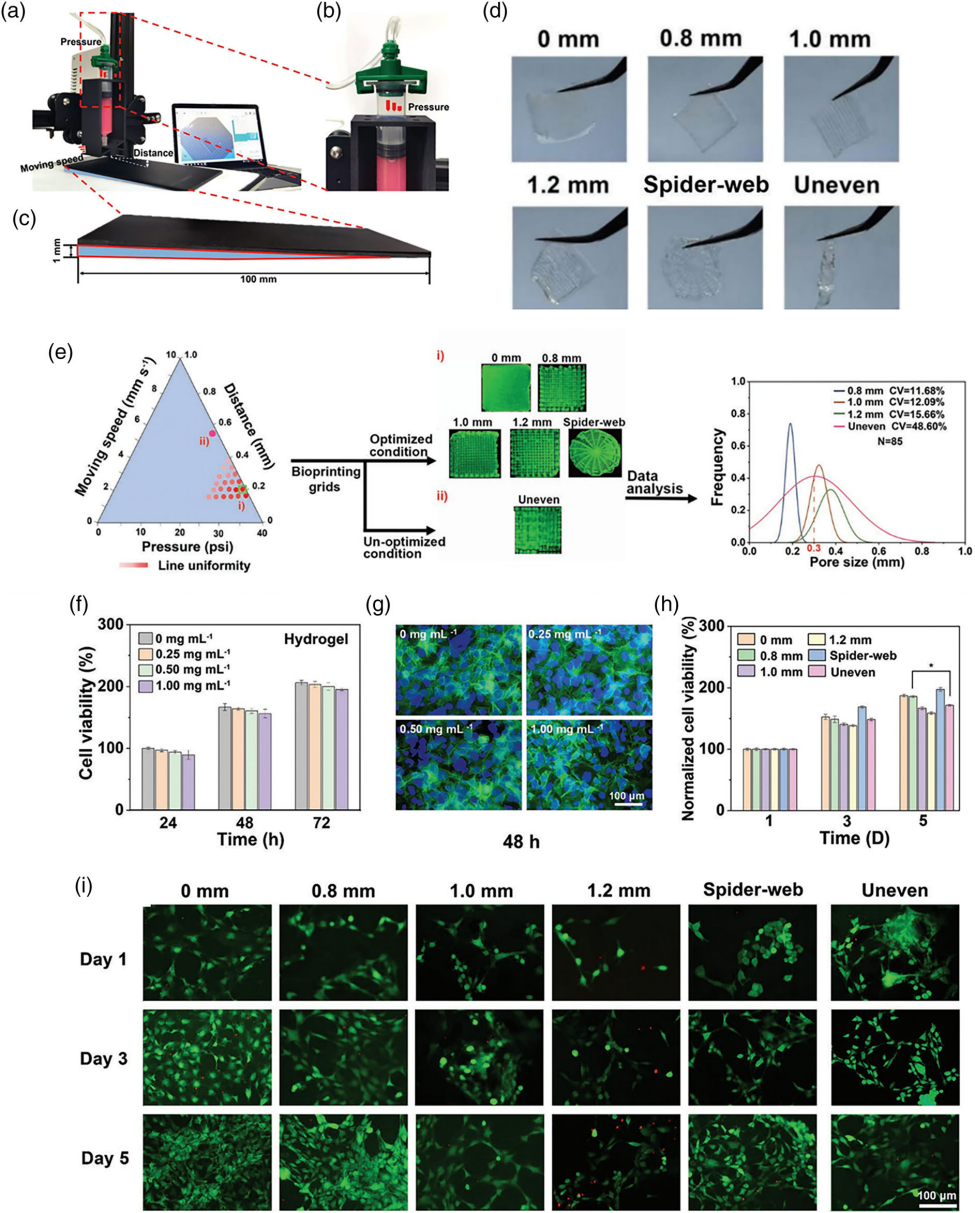
Representative photos show the configuration of the programmable pneumatic extrusion bioprinter as a hardware part of the screening AI‐HTPCSS. (a) The programmable pneumatic extrusion (bio)printer. (b) The modified printhead for the control of extrusion pressure. (c) The graded build plate for the control of the printing distance (i.e., nozzle‐substrate distance). The printing software controlled the speed of the nozzle and the pressure applied to the printhead. (d) Fabrication of multilayer hydrogel scaffolds under optimized and non‐optimized printing conditions. (e) Printing grid‐shaped membranes for diabetic wound healing under optimized and non‐optimized printing conditions. (i) Printed grids under optimized conditions as indicated in the phase diagram. (ii) Printed grids under non‐optimized conditions as indicated in the phase diagram. The distribution curves and their CVs show the pore sizes of the printed grid‐shaped scaffolds with different geometries obtained after image analysis. Frequency is defined as the ratio of the number of occurrences of each pore size to the total number of occurrences of all pore sizes. Pore size refers to the size of pores that are created by the line cross on the surface of the scaffolds. In vitro cellular studies of the printed hydrogel scaffolds. (f) Cell viabilities under different concentrations of the hydrogel‐soaked medium (0, 0.25, 0.50, and 1.00 mg/mL, *p < 0.05, *n* = 3). (g) Immunofluorescence staining images of human epidermal keratinocyte cells treated with different concentrations of the hydrogels (0, 0.25, 0.50, and 1.00 mg/mL) for 48 h. The cytoskeleton was stained green and the nuclei were stained blue. (h) Proliferation of NIH/3T3 fibroblasts on the hydrogel scaffolds (*p < 0.05, *n* = 3). (i) Live/dead staining of NIH/3T3 fibroblasts on the hydrogel scaffolds on Days 1, 3 and 5.^243^

Advanced manufacturing technologies, such as AI‐assisted pneumatic extrusion bioprinting, further refine scaffold architecture. In Figure 15a–i, the influence of printing parameters on scaffold porosity and cell viability is demonstrated, highlighting the uniformity achieved with image‐guided algorithms.^243^

**FIGURE 16:**
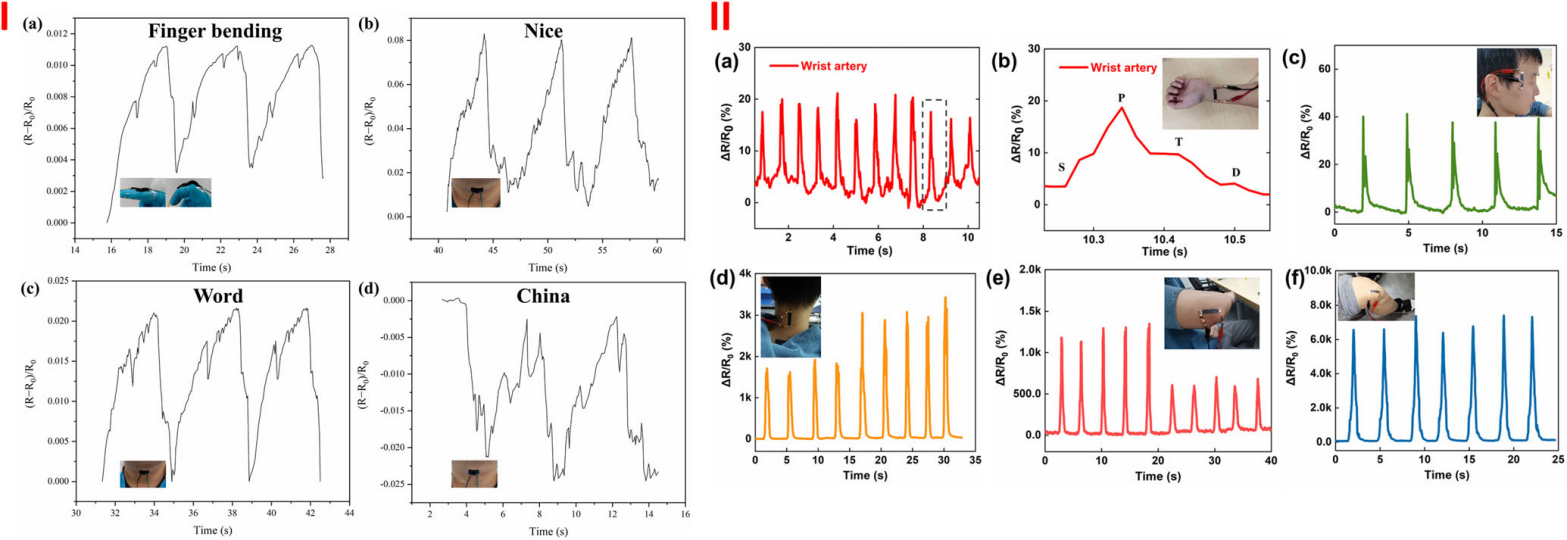
(I) Sensing the signals of encapsulated hydrogel relating to (a) finger bending, (b) the vocalization of the word “Nice,” (c) the vocalization of the word “LOVE,” and (d) the vocalization of the word “China”^244^ (II) The detection of mechanical and physiological signals single‐walled carbon nanotubes crosslinked multilayer graphene (SCG) electronic skin (e‐skin). (a) The detection of pulse signals by the SCG e‐skin on the wrist artery. (b) The zoomed‐in graph of a single pulse signal. Inset: tester with the SCG e‐skin attached to the wrist. (c) The detection of blinking by the SCG e‐skin attached next to the eye. Inset: tester with the SCG e‐skin attached next to the eye. (d) The SCG e‐skin detected small and large ranges of neck movement. Inset: tester with the SCG e‐skin attached to the neck. (e) The SCG e‐skin detected small and large ranges of bending movement of the elbow. Inset: tester with the SCG e‐skin attached to the elbow. (f) Bending movement of the e‐skin. Inset: tester with the SCG e‐skin attached to the knee.^245^

Emerging e‐skin technologies, fabricated from GO, SWCNT, or graphene/PU composites, extend the capabilities of wound dressings to include physiological signal monitoring. Figure 16I,II showcase applications such as muscle movement and pulse detection using e‐skin.^244^, ^245^

These systems are increasingly integrated with AiIoMT platforms for comprehensive data acquisition and real‐time feedback.^226^, ^246^

Nevertheless, significant challenges persist. Regulatory bottlenecks, particularly the absence of harmonized standards for nanotoxicity testing, hamper clinical translation. Moving forward, establishing long‐term safety protocols, including chronic exposure assessments, genotoxicity evaluations, and early‐phase clinical trials, will be pivotal. Collaborative frameworks involving regulatory agencies like the FDA, OECD, and ISO must be prioritized to develop validated testing guidelines.^236^

As noted in Section 5, although many carbon nanomaterial‐based wound dressings show preclinical promise,^247^, ^248^, ^249^, ^250^ none have reached full commercialization. Companies like NanoBioMatters, NanoHealth Solutions, and CarbonMedTech are actively developing platforms based on CNTs, graphene, and carbon‐based nanofibers. The only commercially utilized carbon product remains activated carbon, found in deodorizing dressings such as Actisorb silver 220 and Kocarbon Ag, which are limited to odor absorption.^250^ Clinical adoption will depend on pilot‐scale production, multi‐center trials, and regulatory convergence.

In conclusion, carbon‐based nanomaterials offer unprecedented opportunities to revolutionize wound healing. Through the convergence of material science, smart technologies, AI, and rigorous regulatory oversight, these next‐generation wound dressings can evolve from experimental prototypes into real‐world clinical solutions. Sustained interdisciplinary collaboration is key to unlocking their full translational potential and delivering safe, effective, and personalized wound care therapies.

## 5 COMMERCIALIZED PRODUCTS

In the realm of ongoing research and development, pioneering companies are making significant strides in harnessing the potential of carbon nanomaterials for wound healing. It is important to note that, as of now, no commercialized product in this field has entered the market; rather, all these innovative solutions are currently undergoing clinical trials. Among these, nanofiber dressings incorporating carbon nanotubes or graphene have emerged as promising candidates.^247^, ^248^ These dressings foster wound healing by maintaining a moist environment, enabling targeted drug delivery, and stimulating cell migration and proliferation. Additionally, antimicrobial dressings coated with carbon nanotubes effectively combat bacterial hindrances to healing, eliminating pathogens and creating an optimal environment for the healing process.^249^ Carbon nanomaterials also play a pivotal role in drug delivery, enhancing efficacy while minimizing side effects.^250^

Noteworthy among these developments is the GO‐based wound dressing, crafted from GO hydrogel. This dressing exhibits high conductivity, flexibility, and biocompatibility, absorbing moisture and demonstrating antimicrobial properties for effective wound healing. Another standout product is the nanosphere‐encapsulated heparin wound dressing, employing a hydrogel loaded with heparin‐encapsulated nanospheres. It is important to highlight that these products are still in early development stages, with promising results from preliminary studies hinting at their potential to revolutionize wound healing methodologies. In the dynamic landscape of ongoing research and development, companies like NanoBioMatters, NanoHealth Solutions, and CarbonMedTech are at the forefront of carbon nanomaterial‐based wound care solutions. NanoBioMatters leads in developing solutions with carbon nanotubes and graphene, while NanoHealth Solutions focuses on nanofiber dressings, demonstrating exceptional moisture retention and drug delivery capabilities. CarbonMedTech excels in antimicrobial dressings, particularly with carbon nanotubes, providing robust solutions for optimal healing environments. Currently, the only carbon product that is used in commercial products such as Carbonite, Actisorb silver 220, Cliniflex, Kocarbon Ag, and Asking carbosorb is activated carbon, which is known as activated charcoal and is used in deodorizing dressings to absorb the odor of bacterial and fungal infections.^236^

## 6 CONCLUSION

The skin, as the body's primary protective barrier, is highly susceptible to injuries arising from trauma, infections, chronic conditions, or metabolic disorders such as diabetes. Despite the availability of a wide range of conventional wound dressings—including gauze, hydrocolloids, foams, and hydrogels—the multifaceted biological processes involved in wound healing often render these solutions insufficient, particularly for chronic and non‐healing wounds. Carbon‐based nanomaterials, encompassing graphene‐based materials, carbon nanotubes, carbon dots, NDs, fullerenes, and carbon nano‐onions, offer a transformative platform for wound management due to their multifunctional properties. These include antibacterial activity, antioxidative capacity, immunomodulatory effects, and promotion of angiogenesis and tissue regeneration. This review provides a comprehensive overview of the physicochemical features, therapeutic benefits, and biomedical applications of various classes of carbon‐based nanomaterials in wound healing. However, several critical limitations remain to be addressed before clinical translation can occur. Key among these are toxicity concerns, long‐term biosafety, scalability of synthesis, and the lack of standardized regulatory frameworks. Moreover, differences in animal and human skin physiology complicate the preclinical‐to‐clinical transition, highlighting the need for more representative models and human‐based testing platforms. Recent advances have begun to bridge these gaps. The integration of AI, smart biomaterials, and e‐skin technologies has ushered in a new era of personalized and responsive wound care. AI‐based platforms enable the prediction of healing outcomes, real‐time monitoring of wound environments, and precision delivery of therapeutics. Smart dressings embedded with biosensors and responsive carbon nanostructures are now capable of detecting infection, pH fluctuations, moisture, and inflammation, while dynamically adjusting their therapeutic responses. Despite these promising developments, commercialization remains in its infancy. To move from bench to bedside, concerted efforts are needed across interdisciplinary fields. This includes pilot‐scale manufacturing, well‐structured clinical trials, and international cooperation for risk assessment and regulatory compliance. In conclusion, carbon‐based nanomaterials hold great promise for next‐generation wound healing strategies. With continued advances in nanotechnology, smart sensing, bioprinting, and regulatory science, the path toward safe, effective, and intelligent wound dressings is within reach. The convergence of material science, biomedical engineering, and digital health technologies may ultimately redefine the future landscape of wound care.

## AUTHOR CONTRIBUTION

**Pegah Madaninasab**: conceptualization; investigation; writing—original draft; writing—review and editing. **Mahsa Mohammadzadeh**: Conceptualization; investigation; writing—original draft; writing—review and editing. **Sheyda Labbaf**: Conceptualization; project administration; supervision; writing—review and editing.

## CONFLICT OF INTEREST STATEMENT

The authors declare no conflicts of interest.

## Supporting information


**Movie S1.** The superhydrophobic CNF gauze, applied to the wound for about 2 h, can be easily peeled off without causing tearing of the wound.^175^

**Movie S2.** There is a strong adhesion between the normal gauze and the wound. Peeling off the normal gauze after about 2 h significantly tore the wound and caused secondary bleeding.^175^

## Data Availability

Data sharing not applicable to this article as no data sets were generated or analyzed during the current study.
